# The Complete Genome Sequence of Hyperthermophile *Dictyoglomus turgidum* DSM 6724™ Reveals a Specialized Carbohydrate Fermentor

**DOI:** 10.3389/fmicb.2016.01979

**Published:** 2016-12-20

**Authors:** Phillip J. Brumm, Krishne Gowda, Frank T. Robb, David A. Mead

**Affiliations:** ^1^C5-6 Technologies LLCFitchburg, WI, USA; ^2^DOE Great Lakes Bioenergy Research Center, University of Wisconsin-MadisonMadison, WI, USA; ^3^Lucigen CorporationMiddleton, WI, USA; ^4^Department of Microbiology and Immunology, Institute of Marine and Environmental Technology, University of MarylandBaltimore, MD, USA; ^5^Varigen Biosciences CorporationMadison, WI, USA

**Keywords:** *Dictyoglomus turgidum*, thermophile, biomass degradation, phage, *Dictyoglomi*, DNA polymerase, glucanase, reverse gyrase

## Abstract

Here we report the complete genome sequence of the chemoorganotrophic, extremely thermophilic bacterium, *Dictyoglomus turgidum*, which is a Gram negative, strictly anaerobic bacterium. *D. turgidum* and *D. thermophilum* together form the *Dictyoglomi* phylum. The two *Dictyoglomus* genomes are highly syntenic, and both are distantly related to *Caldicellulosiruptor* spp. *D. turgidum* is able to grow on a wide variety of polysaccharide substrates due to significant genomic commitment to glycosyl hydrolases, 16 of which were cloned and expressed in our study. The GH5, GH10, and GH42 enzymes characterized in this study suggest that *D. turgidum* can utilize most plant-based polysaccharides except crystalline cellulose. The DNA polymerase I enzyme was also expressed and characterized. The pure enzyme showed improved amplification of long PCR targets compared to Taq polymerase. The genome contains a full complement of DNA modifying enzymes, and an unusually high copy number (4) of a new, ancestral family of polB type nucleotidyltransferases designated as MNT (minimal nucleotidyltransferases). Considering its optimal growth at 72°C, *D. turgidum* has an anomalously low G+C content of 39.9% that may account for the presence of reverse gyrase, usually associated with hyperthermophiles.

## Introduction

*Dictyoglomus* species are genetically distinct and divergent from known taxa, and have been assigned to their own phylum, *Dictyoglomi* (Saiki et al., 1985; Euzéby, 2012). They have been cultivated from or detected in anaerobic, hyperthermophilic hot spring environments (Patel et al., 1987; Svetlichny and Svetlichnaya, 1988; Mathrani and Ahring, 1991; Kublanov et al., 2009; Gumerov et al., 2011; Kochetkova et al., 2011; Burgess et al., 2012; Sahm et al., 2013; Coil et al., 2014; Menzel et al., 2015) or isolated from paper-pulp factory effluent (Mathrani and Ahring, 1992), but only two *Dictyoglomus* species have been validly described in the literature (Saiki et al., 1985; Svetlichny and Svetlichnaya, 1988). Both strains grow up to 80°C, are Gram negative, and exhibit unusual morphologies consisting of filaments, bundles, and spherical bodies. The first described *Dictyoglomus* species, *Dictyoglomus thermophilum* was isolated from Tsuetate Hot Spring in Kumamoto Prefecture, Japan (Saiki et al., 1985). The genome of *D. thermophilum* has been sequenced (Coil et al., 2014), and a number of potentially useful enzymes including amylase (Fukusumi et al., 1988; Horinouchi et al., 1988), xylanases (Gibbs et al., 1995; Morris et al., 1998), a mannanase (Gibbs et al., 1999) and an endoglucanase (Shi et al., 2013) have been cloned and characterized. The second described species, *Dictyoglomus turgidus*, was isolated from a hot spring in the Uzon Caldera, in eastern Kamchatka, Russia (Svetlichny and Svetlichnaya, 1988). The name *Dictyoglomus turgidus* was subsequently corrected to *Dictyoglomus turgidum* (Euzéby, 1998). Unlike *D. thermophilum, D. turgidum* was reported to grow on a wide range of substrates including starch, cellulose, pectin, carboxymethylcellulose, lignin, and humic acids, but not on pentose sugars such as xylose and arabinose (Svetlichny and Svetlichnaya, 1988). Because of the wide range of substrates utilized, *D. turgidum* was selected for enzyme library construction and carbohydrase screening (Brumm et al., 2011) as well as whole genome sequencing. Here we describe the complete genome sequence of *D. turgidum*, bioinformatic analysis of the metabolism of this unusual organism, and comparative analysis with the genome of *D. thermophilum*. We also present functional analysis of its DNA Pol I gene and a number of novel carbohydrases.

## Materials and methods

*D. turgidum* strain 6724^T^ was obtained from the Deutsche Sammlung von Mikroorganismen und Zellkulturen GmbH (DSMZ). 10G electrocompetent *E. coli* cells, pEZSeq (a lac promoter vector), Taq DNA polymerase and OmniAmp DNA polymerase were obtained from Lucigen, Middleton, WI. Azurine cross-linked-labeled polysaccharides were obtained from Megazyme International (Wicklow, Ireland). 4-methylumbelliferyl-β-D-cellobioside (MUC), 4-methylumbelliferyl-β-D -xylopyranoside (MUX), and 4-methylumbelliferyl-β-D- glucoyranoside (MUG) were obtained from Research Products International Corp. (Mt. Prospect, IL). CelLytic IIB reagent, pNP-β-glucoside, pNP-β-cellobioside, 4-methylumbelliferyl-α-D-arabinofuranoside (MUA), 4-methylumbelliferyl-β-D-lactopyranoside (MUL), 5-Bromo-4-chloro-3-indolyl α-D-galactopyranoside (X-α-Gal, XAG), and 5-Bromo-4-chloro-3-indolyl β-D-galactopyranoside (X-gal, XG) were purchased from Sigma-Aldrich (St. Louis, MO). All other chemicals were of analytical grade.

*D. turgidum* DSM 6724™ was obtained from the DSMZ culture collection and maintained on DSM Medium 516 reduced with Na_2_S and N_2_ at 75°C in Balch tubes with a headspace of N_2_. Cultures grown in 1 L stoppered flasks were harvested for DNA preparation. YT plate media (16 g/l tryptone, 10 g/l yeast extract, 5 g/l NaCl and 16 g/l agar) was used in all molecular biology screening experiments. Terrific Broth (12 g/l tryptone, 24 g/l yeast extract, 9.4 g/l K_2_HPO_4_, 2.2 g/l KH_2_PO_4_, and 4.0 g/l glycerol added after autoclaving) was used for liquid cultures.

A cell concentrate of *D. turgidum* strain 6724™ was lysed using a combination of SDS and proteinase (Sambrook et al., 1989) and genomic DNA was purified using phenol/chloroform extraction. The genomic DNA was precipitated, treated with RNase to remove residual contaminating RNA, and fragmented by hydrodynamic shearing (HydroShear apparatus, GeneMachines, San Carlos, CA) to generate fragments of 2–4 kb. The fragments were purified on an agarose gel, end-repaired, and ligated into pEZSeq (Lucigen Corp., Middleton WI). The recombinant plasmids were then used to transform electrocompetent cells. A copy of the library containing the *Dictyoglomus turgidum* genomic DNA was submitted to the Joint Genome Institute of the Department of Energy for whole genome sequencing; a second copy of the library was used for carbohydrase screening experiments.

The genome of *D. turgidum* DSM 6724™ was sequenced at the Joint Genome Institute (JGI) using a combination of 3 and 8 kb DNA libraries. In addition to 20x Sanger sequencing, 454 pyrosequencing was done to a depth of 20x coverage. Draft assemblies were based on 32,817 total reads. The Phred/Phrap/Consed software package was used for sequence assembly and quality assessment (Ewing and Green, 1998; Gordon et al., 1998). After the shotgun stage, reads were assembled with parallel phrap. Possible mis-assemblies were corrected with Dupfinisher or transposon bombing of bridging clones. Gaps between contigs were closed by editing in Consed, custom primer walking or PCR amplification. A total of 80 additional reactions were necessary to close gaps and to raise the quality of the finished sequence. The completed genome sequence of *D. turgidum* DSM 6724™ contains 34,756 reads, achieving an average of 17.3x coverage. The Accession number for the complete genome is NC_011661.

Genes were identified using Prodigal (Hyatt et al., 2010) as part of the Oak Ridge National Laboratory genome annotation pipeline, followed by a round of manual curation using the JGI GenePRIMP pipeline. The predicted CDSs were translated and used to search the National Center for Biotechnology Information (NCBI) nonredundant database, UniProt, TIGRFam, Pfam, PRIAM, KEGG, COG, and InterPro databases. These data sources were combined to assert a product description for each predicted protein. Non-coding genes and miscellaneous features were predicted using tRNAscan-SE (Lowe and Eddy, 1997), RNAMMer (Lagesen et al., 2007), Rfam (Griffiths-Jones et al., 2003), TMHMM (Krogh et al., 2001), CRISPRFinder (Grissa et al., 2007), and signalP (Krogh et al., 2001). RAST annotations (Aziz et al., 2008) of *D. turgidum* and *D. thermophilum* were carried out in parallel to further clarify genomic relationships using SEED genome comparison tools (Overbeek et al., 2005).

The phylogeny of *D. turgidum* was determined using its 16S ribosomal RNA (rRNA) gene sequence as well as those of the most closely related 16S rRNA sequences identified by BLASTn. 16S rRNA gene sequences were aligned using MUSCLE (Edgar, 2004), pairwise distances were estimated using the maximum composite likelihood (MCL) approach, and initial trees for heuristic search were obtained automatically by applying the neighbor-joining method in MEGA7 (Kumar et al., 2016). The alignment and heuristic trees were then used to infer the phylogeny using the maximum likelihood method based on Tamura-Nei (Tamura and Nei, 1993; Tamura et al., 2011). The phylogeny of the reverse gyrase protein sequence was inferred using the Neighbor-Joining method. The optimal tree with the sum of branch length = 1.99686421 is shown. The percentage of replicate trees in which the associated taxa clustered together in the bootstrap test (1000 replicates) are shown next to the branches. The tree is drawn to scale, with branch lengths in the same units as those of the evolutionary distances used to infer the phylogenetic tree. The evolutionary distances were computed using the Maximum Composite Likelihood method and are in the units of the number of base substitutions per site. The analysis involved 7 nucleotide sequences. Codon positions included were 1st+2nd+3rd+Noncoding. All positions containing gaps and missing data were eliminated. There were a total of 3230 positions in the final dataset. Evolutionary analyses were conducted in MEGA7 (Kumar et al., 2016).

The *endo*-glucanase specificity of enzymes was determined in 0.50 ml of 50 mM acetate buffer, pH 5.8, containing 0.2% azurine cross-linked-labeled (AZCL) insoluble substrates and 50 μl of clarified lysate. Each purified enzyme was evaluated for *endo*-activities using the following set of substrates: AZCL-arabinan (AR), AZCL-arabinoxylan (AX), AZCL-β-glucan (BG), AZCL-curdlan (CU), AZCL-galactan (GL), AZCL-galactomannan (GM), AZCL-hydroxyethyl cellulose (HEC), AZCL-pullulan (PUL), AZCL-rhamnogalacturonan (RH), and AZCL-xyloglucan (XG). Assays were performed at 70°C, with shaking at 1000 rpm, for 60 min in a Thermomixer R (Eppendorf, Hamburg, Germany). Tubes were clarified by centrifugation and absorbance values at 600 nm determined using a Bio-Tek EL_x_800 plate reader. The exo-glucanase specificity of enzymes was determined by spotting 2.0 μl of clarified lysate directly on agar plates containing 10 mM 4-methylumbelliferyl substrate. Plates were placed in a 70°C incubator for 60 min and then examined using a hand-held UV lamp and compared to negative and positive controls for fluorescence.

Amplification efficacy was compared between Dtur, Taq and OmniAmp DNA polymerases (DNAP) in side by side PCR reactions using four different sized amplicons (0.9, 2.8, 5.0, and 10.0 Kb). PCR reaction conditions contained 1–20 ng of template DNA, 2.5U of Taq DNAP or 5U Dtur or OmniAmp DNAP (Lucigen Corp.), 200 μM dNTPs, and 0.5 μM primers in a 50 μl reaction. DNAP buffer (1X) contained10 mM Tris-HCl (pH 8.8), 10 mM KCl, 10 mM NH2SO4, 2 mM MgSO4, 0.1% tritonX-100, and 15% sucrose. Cycling conditions were 94°C 2 min and 30 cycles of 94°C for 15 s, 60°C for 30 s, and 72°C for 1 min per kb. The templates and PCR primers are as follows: pUC19 0.9 kb amplicon primers (CCC CTA TTT GTT TAT TTT TCT AAA ATT CAA TAT GTA TCC GCT and TTA CCA ATG CTT AAT CAG TGA GGC ACC TAT CT), *E. coli* 2.8 kb amplicon primers (TAC TGT CTG CCA TGG TTC AGA TCC CCC AAA ATC CAC TTA TCC TTG TAG A and TTA TCT GTG GTC GAC TTA GTG CGC CTG ATC CCA GTT TTC GCC ACT CCC CA), *E. coli* 5 kb amplicon primers (TCT CTC CGA CCA AAG AGT TG and GAA ACA TTG AGC GAA GAG GA), and *E. coli* 10 kb amplicon primers (CTA TGA TTA TCT AGG CTT AGG GTC AC and CAG TGT AGA GAG ATA GTC AGG AGT TA).

Functional screening for active carbohydrase enzymes involved plating transformed *E. coli* cells containing 2–4 kb Dtur genomic DNA inserts in the pEZSeq vector on YT agar containing IPTG (for *lac*Z promoter induction) and one of the fluorescent substrates MUC, MUG or MUX. A long wavelength UV lamp was used to locate colonies that were fluorescent, which were sequenced by Sanger chemistry to identify the gene. Genes identified in the functional screen as well as additional genes of interest from the completed genome were amplified without their respective signal sequence, ligated into pET28A, and transformed into BL21(DE3) *E. coli* competent cells. Recombinant clones were cultured overnight at 37°C, 100 rpm, in 100 ml Luria Broth containing 50 mg/l kanamycin. Expression was induced using 1 mM IPTG, and cultures were harvested 18 h after induction. Cells were pelleted by centrifugation, and the pellets were lysed using Cellytic B reagent. Proteins were purified using standard methods for His-tagged proteins (Spriestersbach et al., 2015), and their purity and identity verified by SDS PAGE.

*D. turdigum* DNA polymerase I (Dtur DNAP) was cloned by PCR amplification using the proofreading enzyme Phusion (NEB, Waltham MA) and forward and reverse 24 base oligonucleotides that spanned the start and stop codons. The amplified DNA was inserted into the rhamnose promoter vector pRham containing an N terminal histidine tag and transformed into 10G competent *E. coli* cells (Lucigen Corp.). Recombinant Dtur DNAP production was induced by rhamnose and the enzyme was purified using standard methods for His-tagged proteins (Spriestersbach et al., 2015).

## Results

### Genome of *D. turgidum*

**Figure 1:**
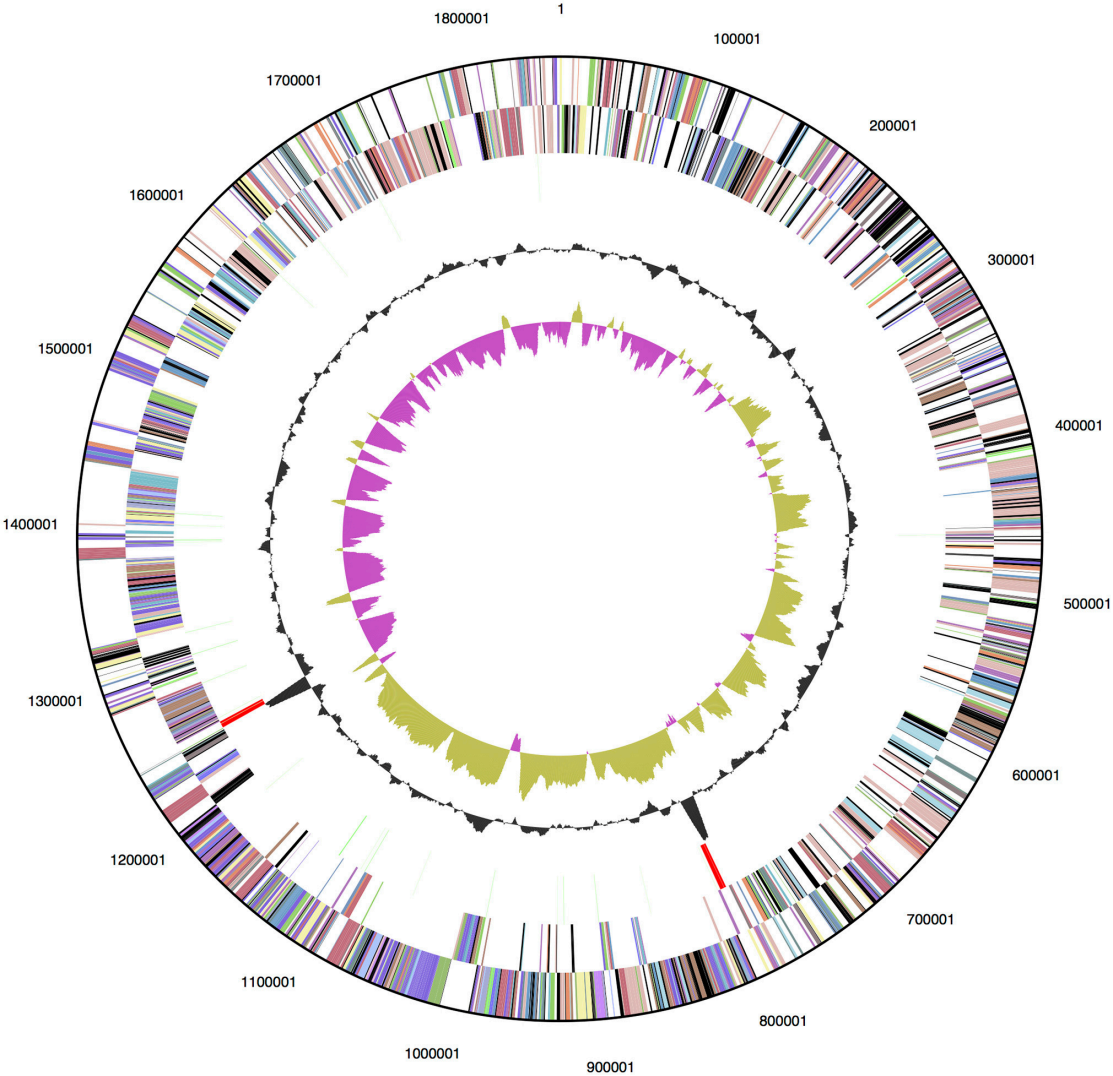
**Genome map of ***D. turgidum*****. From outside to the center: genes on forward strand (color by COG categories); genes on reverse strand (color by COG categories); RNA genes (tRNAs green, rRNAs red, other RNAs black); GC content; GC skew.

The genome of *D. turgidum* DSM 6724™ consists of a single chromosome of 1,855,560 bp and no plasmids or extrachromosomal elements. The GC content of the chromosome is 33.96% based on the genome sequence, slightly higher than the reported value of 32.5% (Svetlichny and Svetlichnaya, 1988) and is predicted to contain 1813 protein-coding genes and 52 RNA genes (Figure 1). The completed genome sequence is available from GenBank (GenBank: CP001251.1). Based on 16S rRNA gene sequence analysis, *D. turgidum* DSM 6724 and *D. thermophilum* are separate species. This is confirmed by average nucleotide analysis (ANI), where *D. turgidum* and *D. thermophilum* are calculated to have 82.4% average nucleotide identity, below the threshold for members of the same species.

Of the 1813 protein-coding genes, 1354 genes (72.6%) were assigned to COGs categories (Table 1). The fraction of the genes annotated as members of COG class G, carbohydrate transport and metabolism (highlighted in bold), 13.4%, is greater than the fraction observed for 95% of genomes in the MicrobesOnline database (Dehal et al., 2010). This represents the lower limit of proteins involved in carbohydrate metabolism, because it does not include any proteins in categories R, S or not in COGS that were not identified by the algorithm as being involved in carbohydrate metabolism. A number of pectate lyases, for example, are not identified as members of COGs class G. No other COGs category had a significantly higher than average number of members, and no COGs category had a significantly lower than average percentage of members.

**Table 1:** **Number of genes associated with general COG functional categories**.

**Code**	**Value**	**Percentage**	**Description**
J	168	11.0%	Translation, ribosomal structure and biogenesis
K	76	5.0%	Transcription
L	61	4.0%	Replication, recombination and repair
B	1	0.1%	Chromatin structure and dynamics
D	19	1.2%	Cell cycle control, Cell division, chromosome partitioning
V	40	2.6%	Defense mechanisms
T	48	3.1%	Signal transduction mechanisms
M	87	5.7%	Cell wall/membrane biogenesis
N	20	1.3%	Cell motility
U	18	1.2%	Intracellular trafficking and secretion
O	61	4.0%	Posttranslational modification, protein turnover, chaperones
C	79	5.2%	Energy production and conversion
**G**	**205**	**13.4%**	**Carbohydrate transport and metabolism**
E	170	11.1%	Amino acid transport and metabolism
F	60	3.9%	Nucleotide transport and metabolism
H	73	4.8%	Coenzyme transport and metabolism
I	44	2.9%	Lipid transport and metabolism
P	77	5.0%	Inorganic ion transport and metabolism
Q	18	1.2%	Secondary metabolites biosynthesis, transport and catabolism
R	130	8.5%	General function prediction only
S	58	3.8%	Function unknown
–	511	27.4%	Not in COGs

*Highlighted in bold, COG class G. The fraction of the genes annotated as members of this class is greater than the fraction observed for 95% of genomes in the MicrobesOnline database*.

### Genomic insights into the relationship of *D. turgidum* to *D. thermophilum* and other organisms

While being separate species, an in-depth comparison of the two *Dictyoglomi* genomes shows that *D. turgidum* is closely related to *D. thermophilum* on a number of levels. The genomes are similar in size, with *D. turgidum* being slightly smaller than the genome of *D. thermophilum* (1,855,560 bp vs. 1,959,987 bp) and containing approximately 100 fewer protein coding genes (1813 vs. 1912). The two organisms have a highly conserved set of genes present in their genomes. Over 95% of the proteins present in *D. turgidum* have orthologs in *D. thermophilum*. There are only 43 proteins of greater than 100 amino acids present in *D. turgidum* without orthologs in *D. thermophilum*, and there are only 109 proteins of greater than 100 amino acids present in *D. thermophilum* without orthologs in *D. turgidum*. Of the proteins with orthologs in both species, there are 614 proteins with >90% sequence identity.

**Figure 2:**
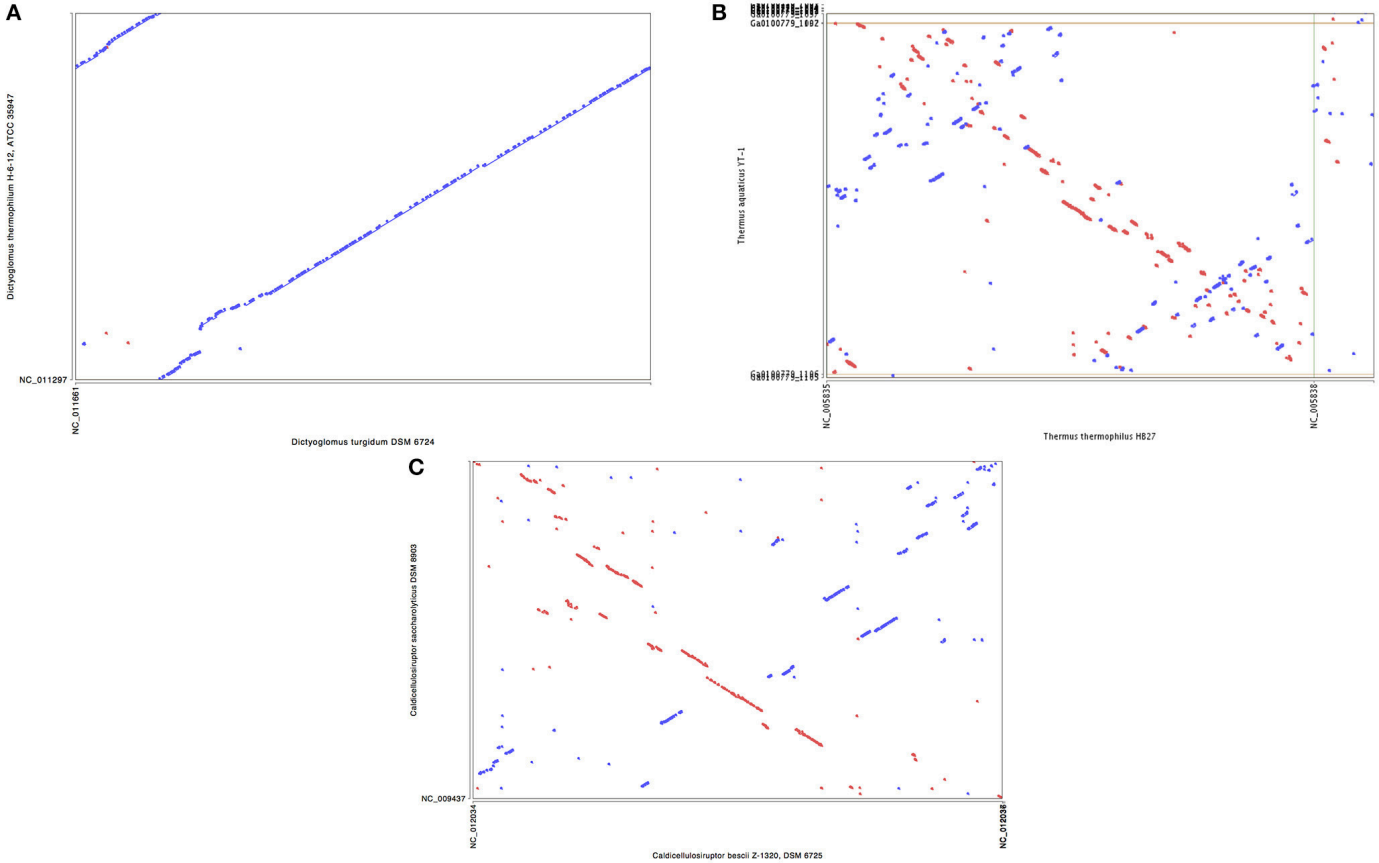
**Synteny plot of selected genomes**. MUMmer (Delcher et al., 2003) was used to generate the dotplot diagram between sets of two genomes. The six frame amino acid translation of the DNA input sequences were used for comparing genomes using PROmer software. Clockwise from top **(A)** genomes of *D. turgidum* and *D. thermophilum*; **(B)** genomes of *T. thermophilus* and *T. aquaticus*; **(C)** genomes of *C. bescii* and *C. saccharolyticus*.

Synteny plots were generated using both RAST and IMG annotation methods. The two annotation methods gave essentially identical plots, as did plots based on DNA or protein sequences. The plots show the genomes of *D. turgidum* and *D. thermophilum* have highly conserved large and small-scale organization (Figure 2A). This conserved organization appears to be an unusual phenomenon. Two sets of thermophilic organisms with similar ANI values, *T. thermophilus* and *T. aquaticus* (84.3% ANI, Figure 2B) and *C. bescii* and *C. saccharolyticus* (82.0% ANI, Figure 2C) show only limited short-range synteny and no extensive long-range synteny. It is unclear if this conserved genomic organization is limited to these two species, or is present in all *Dictyoglomi* genomes.

**Figure 3:**
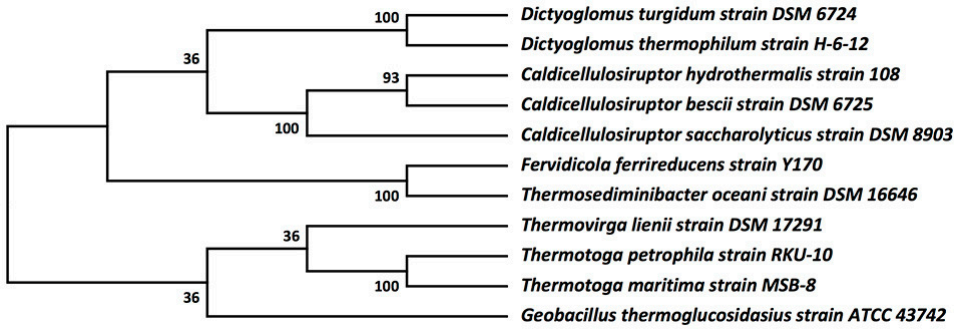
**Molecular phylogenetic analysis of ***Dictyoglomus turgidum*** using 16S rDNA sequences**. Molecular phylogenetic analysis by Maximum Likelihood method was detailed in the Material and Methods Section. The bootstrap consensus tree inferred from 550 replicates [2] is taken to represent the evolutionary history of the taxa analyzed. Branches corresponding to partitions reproduced in less than 50% bootstrap replicates are collapsed. The percentage of replicate trees in which the associated taxa clustered together in the bootstrap test (550 replicates) are shown next to the branches. Sequences used for the analysis are: *Dictyoglomus turgidum* strain DSM 6724; NR_074885; *Dictyoglomus thermophilum* strain H-6-12, NR_029235.1; *Fervidicola ferrireducens* strain Y170, NR_044504.1; *Thermosediminibacter oceani* strain DSM 16646; NR_074461.1; *Caldicellulosiruptor saccharolyticus* strain DSM 8903; NR_074845.1; *Caldicellulosiruptor hydrothermalis* strain 108, NR_074767.1; *Caldicellulosiruptor bescii* strain DSM 6725; NR_074788.1; *Desulfotomaculum kuznetsovii* strain DSM 6115; NR_075068.1; *Thermovirga lienii* strain DSM 17291; NR_074606.1; *Thermotoga petrophila* strain RKU-10, NR_042374.1; *Thermotoga naphthophila* strain RKU-10, NR_112092.1; *Thermotoga maritima* strain MSB-8, NR_029163.1; and *Geobacillus thermoglucosidasius* strain ATCC 43742; NR_112058.1.

The relationship of these two *Dictyoglomus* species to other organisms appears significantly more complicated, depending on the type of analysis and interpretation (Love et al., 1993; Rees et al., 1997; Takai et al., 1999; Ding et al., 2000; Wagner and Wiegel, 2008). Phylogenetic analysis using 16S rRNA shows the two *Dictyoglomus* species appear most closely related to *Thermotoga* species before bootstrapping (data not shown). After bootstrapping, the relationship shifts dramatically, with the two *Dictyoglomus* species becoming most closely related to *Caldicellulosiruptor* species (Figure 3). Previous work using average nucleotide identity (ANI) calculations (Nishida et al., 2011) identified *Thermotoga* species as the closest relatives to *Dictyoglomus*. ANI values were generated using the *D. thermophilum* genome, eight finished, closed *Thermotoga* genomes and three finished, closed *Caldicellulosiruptor* genomes. ANI values (Kim et al., 2014) were computed as pairwise bidirectional best nSimScan hits of genes having 70% or more identity and at least 70% coverage of the shorter gene. ANI calculations performed as described above yielded 82.4% identity between the genomes of *D. turgidum* and *D. thermophilum*, based on 1584 proteins (87% of the genome) that met the criteria. The value of 82.4% is well below the cut-off value of 98% for strains of the same species, and confirms that *D. turgidum* and *D. thermophilum* are separate species. The ANI calculations found 67–68% identity between *D. turgidum* and the three *Caldicellulosiruptor* species, based on 124–129 proteins per genome that met the criteria for the calculation (approximately 7% of the genome). ANI calculations found 66–68% identity between *D. turgidum* and the eight *Thermotoga* species, based on the 36–64 proteins per genome that met the criteria (approximately 2–4% of the genome). Rather than identifying relationships among these organisms, the low number of proteins in *D. turgidum* with at least 70% identity to the proteins in these 11 strains (on which these ANI values are calculated) further demonstrates the uniqueness of this organism.

### Protein and amino acid metabolism

Based on the MEROPS database (Rawlings et al., 2014), the *D. turgidum* genome codes for 55 potential peptidases. This value is within the range of peptidases reported in the database for *Thermotoga* species (52–67) and *Caldicellulosiruptor* species (54–74). Of the 55 potential peptidases, only a single peptidase, Dtur_0603, possesses an annotated signal sequence and is predicted to be secreted. While possessing only a single secreted peptidase to generate amino acids and peptides, *D. turgidum* possesses nine potential membrane transporter systems to transport amino acids and peptides into the cell. These nine transporters include seven annotated oligopeptide/dipeptide ABC transporter systems (Dtur_0082 through Dtur_0086; Dtur_0158 through Dtur_0162; Dtur_0214 through Dtur_0217; Dtur_0664 through Dtur_0668; Dtur_1061 through Dtur_1064; Dtur_1704 and Dtur_1707; Dtur_1719 through Dtur_1722) as well as two amino acid ABC transporter systems (Dtur_1051 through Dtur_1053 and Dtur_0932 through Dtur_0936).

*D. turgidum* appears to utilize the amino acids and peptides taken up for protein synthesis, but it is unable to metabolize most amino acids as an energy or carbon source. Based on the BioCyc (Karp et al., 2005; Caspi et al., 2014) and SEED (Devoid et al., 2013) metabolic reconstructions from the genome sequence, *D. turgidum* is lacking degradation pathways for the following 13 amino acids: aspartate, asparginine, cysteine, histidine, isoleucine, leucine, lysine, phenylalanine, proline, serine, tryptophan, tyrosine, and valine. Arginine is not metabolized, but may be converted to putrescine.

Only four amino acids appear to be metabolized by *D. turgidum*. Glutamate is converted to methyl aspartate using glutamate mutase (Dtur_1345 through Dtur_1347) and then to pyruvate and acetate. Threonine can be degraded to glycine and acetaldehyde via threonine aldolase (Dtur_0449), and the acetaldehyde generated is then converted to acetyl-CoenzymeA (acetyl-CoA) via aldehyde dehydrogenase (Dtur_0484). Alanine can be converted to pyruvate by alanine dehydrogenase (Dtur_1049), and glycine can be converted to ammonium 5,10-methylenetetrahydrofolate via glycine dehydrogenase and glycine cleavage system T protein (Dtur_1515 through Dtur_1518). The ability to utilize these four amino acids may be responsible for the observation of growth by *D. turgidum* on yeast extract, peptone, and casamino acids (Svetlichny and Svetlichnaya, 1988).

### Monosaccharide metabolism

Based on the genomic reconstruction of Dtur, the organism is able to metabolize most five and six carbon sugars, and the following pathways are predicted. Arabinose is utilized via isomerization to L-ribulose (Dtur_0379, or other isomerase), phosphorylation by L-ribulose kinase (Dtur_1748) to L-ribulose-5-phosphate, and isomerization by L-ribulose-5-phosphate-4-epimerase (Dtur_1734) to D-xylulose-5-phosphate, which is then metabolized via the pentose phosphate pathway. Rhamnose is utilized via isomerization by L-rhamnose isomerase to L-rhamulose (Dtur_0427), phosphorylation by L-rhamulose kinase (Dtur_1748) to L-rhamulose-1-phosphate, and cleavage into dhihydroxyacetone phosphate and L-lactaldehyde. Xylose is utilized via isomerization by xylose isomerase (Dtur_0036 or other sugar isomerase) to xylulose, and the xylulose is phosphorylated by xylulose kinase to (Dtur_0920) to D-xylulose-5-phosphate, which is then metabolized via the pentose phosphate pathway.

Fucose is utilized via isomerization by L-fucose isomerase to L-fuculose (Dtur_0410), phosphorylation by L-fuculokinase (Dtur_0920) to L-fuculose-1-phosphate, and cleavage into dhihydroxyacetone phosphate and L-lactaldehyde. Galactose is phosphorylated by galactose kinase (Dtur_1195) to galactose-1-phosphate, which is converted to UDP-galactose by galactose-1-phosphate uridyl transferase (Dtur_1196), isomerized by UDP-glucose-4-epimerase (Dtur_1352) to UDP-glucose, and finally to glucose-1-phosphate by UTP-glucose-1-phosphate uridylyltransferase (Dtur_1627). Mannose is phosphorylated by mannose kinase (Dtur_0176; Dtur_0716 or other annotated sugar kinase) to generate mannose-1-phosphate. The mannose-1-phosphate is isomerized to mannose-6-phosphate by phosphomannomutase/phosphoglucomutase (Dtur_0067) and then to fructose-6-phosphate by phosphoglucose/phosphomannose isomerase (Dtur_1271). UDP-glucose is either isomerized to fructose, or oxidized to UDP-glucuronate using either Dtur_575 or Dtur_718. The UDP-glucuronate can then be further oxidized to ribulose-5-phosphate by 6-phosphogluconate dehydrogenase (Dtur_0197).

Galacturonate generated by pectin degradation may be epimerized by one of the six UDP sugar epimerase genes found in the genome. Rarely-encountered sugars may be handled by any of a number of sugar isomerases. Dtur rhamnose isomerase (Dtur_0427) isomerizes seven monosaccharides: L-rhamnose, L-lyxose, L-mannose, L-xylulose, L-fructose, D-allose, and D-ribose (Kim et al., 2013). The Dtur fucose isomerase (Dtur_0410) isomerizes L-fucose, D-arabinose, D-altrose, and L-galactose (Hong et al., 2012). Dtur also possesses a cellobiose 2-epimerase that may isomerize non-metabolized disaccharides into easily-degradable ones (Kim et al., 2012).

### Polysaccharide degradation and transport

Polysaccharide degradation by *D. turgidum* is of interest for a number of reasons. Analysis of the *D. turgidum* genome shows an enrichment in COGS family members annotated as involved in carbohydrate transport and metabolism (Table 1). *D. turgidum* is reported to utilize polysaccharides such as starch, cellulose, pectin, glycogen, and carboxymethyl cellulose (Svetlichny and Svetlichnaya, 1988) while *D. thermophilum* is reported to utilize starch, but not cellulose. Finally, a number of carbohydrates with potential industrial applications have been identified in the two *Dictyoglomus* species including amylases and xylanases. A combination of genomic and enzymatic analyses was carried out to clarify the polysaccharide degradation potential of *D. turgidum*.

Analysis of the *D. turgidum* genome reveals a wide range of genes coding for annotated extracellular and intracellular polysaccharide degrading enzymes. The CAZy database (Lombard et al., 2014) identifies 57 glycosyl hydrolases (GH), 3 polysaccharide lyases (PL) and 6 carbohydrate esterases (CE) in the Dtur genome. Based on signal sequence predictions (Petersen et al., 2011), 20 of the polysaccharide-degrading enzymes are secreted into the medium (Table 2), where they degrade polysaccharides into oligosaccharides and monosaccharides. After polysaccharide degradation, 18 annotated three-component ABC carbohydrate transporters are predicted to transport monosaccharides and oligosaccharides into the cell. *D. turgidum* is reported to utilize fructose, glucose, rhamnose, inositol, mannitol, and sorbitol (Svetlichny and Svetlichnaya, 1988), indicating ABC carbohydrate transporters exist for these monosaccharides and sugar alcohols. *D. turgidum* cannot utilize arabinose, fucose, galactose, mannose, or xylose, indicating a lack of dedicated transport systems for these monosaccharides. These sugars may be transported into the cell as oligosaccharides by the oligosaccharide transporters and degraded to monosaccharides in the cytoplasm. Once inside the cell, oligosaccharides are degraded into monosaccharides by a combination of 46 *exo*-acting and *endo*-acting enzymes (Table 3). Working together, these 46 enzymes appear capable of degrading oligosaccharides from most plant-based polysaccharides to monosaccharides.

**Table 2:** **Annotated secreted polysaccharide-degrading enzymes**.

**Gene**	**GH family**	**Annotated activity**	**Nearest ortholog**	**Identity**
Dtur_0097	GH 44	β-mannanase	Calkro_0851	70.1%
Dtur_0172	GH 28	pectinase	Cphy_3310	47.1%
Dtur_0243	GH 11	xylanase	Calkro_0081	83.7%
Dtur_0276	GH 5	cellulase	Mahau_0466	59.9%
Dtur_0277	GH 26	β-mannanase	BG52_11385	52.7%
Dtur_0430	PL 1	pectate lyase	SNOD_03765	42.1%
Dtur_0431	PL 1	pectate lyase	M769_0111315	60.1%
Dtur_0432	PLNC	pectate lyase	CSE_02370	57.3%
Dtur_0433	CE 8	pectin esterase	Calkro_0154	56.0%
Dtur_0628	GH 12	curdlanase	CTN_1107	48.4%
Dtur_0669	GH 5	cellulase	Mahau_0466	54.7%
Dtur_0675	GH 57	α-amylase	ANT_11030	41.3%
Dtur_0676	CBM9	α-amylase	COCOR_00322	39.7%
Dtur_0857	GH 53	β-galactanase	TRQ7_08325	56.5%
Dtur_1586	GH 5	cellulase	BSONL12_10711	41.5%
Dtur_1675	GH 13	α-amylase	CAAU_0986	51.6%
Dtur_1715	GH 10	xylanase	Pmob_0231	46.9%
Dtur_1729	GH 43	β-xylosidase	Csac_1560	67.9%
Dtur_1739	GH 51	β-xylosidase	Calhy_1625	58.9%
Dtur_1740	GH 39	β-xylosidase	TRQ7_03440	38.3%

**Table 3:** **Annotated intracellular polysaccharide-degrading enzymes**.

**Gene**	**GH family**	**Annotated activity**	**Nearest ortholog**	**Identity**
Dtur_0081	GH 2	β-galactosidase	Calhy_1828	60.9%
Dtur_0157	GH 4	α-glucosidase	Mc24_02443	47.5%
Dtur_0171	GH 31	α-glucosidase	A500_11654	44.9%
Dtur_0219	GH 3	β-glucosidase	*D. tunisiensis* bglB3	67.3%
Dtur_0222	GH 20	β-hexosaminidase	CDSM653_01797	67.2%
Dtur_0242	CE NC	feruloyl esterase	TM_0033	55.1%
Dtur_0265	CE 7	acetyl xylan esterase	Tmari_0074	66.4%
Dtur_0289	GH 3	β-glucosidase	Cst_c03130	66.8%
Dtur_0315	GH 29	α-fucosidase	Tthe_0662	60.7%
Dtur_0320	GH 31	α-glucosidase	Csac_1354	65.9%
Dtur_0321	GH 3	β-glucosidase	Cst_c12090	50.1%
Dtur_0384	GH 4	α-glucosidase	CTER_5006	48.4%
Dtur_0435	PL 1	pectate lyase	MB27_42800	36.0%
Dtur_0440	GH 4	α-galacturonidase	BTS2_1711	61.6%
Dtur_0450	CE 4	deacetylase	Tnap_0743	67.4%
Dtur_0451	GH 16	curdlanase	TRQ7_04835	50.9%
Dtur_0462	GH 1	β-glucosidase	CLDAP_02840	48.5%
Dtur_0490	GH 31	α-glucosidase	Tbis_2416	45.4%
Dtur_0502	GH 127	β-L-arabinofuranosidase	CTN_0404	56.3%
Dtur_0505	GH 42	β-galactosidase	Mahau_1293	59.2%
Dtur_0523	GH 18	chitinase	Bccel_2454	50.1%
Dtur_0551	GH 32	invertase	Calhy_2186	47.6%
Dtur_0629	GH 26	β-mannanase	Calkro_1144	54.5%
Dtur_0650	GH 31	α-glucosidase	TheetDRAFT_1156	45.2%
Dtur_0658	GH 130	α-D-mannosyltransferase	X274_02975	41.2%
Dtur_0670	GH 5	cellulase	Mahau_0466	61.7%
Dtur_0671	GH 5	cellulase	TM_1752	58.7%
Dtur_0770	GH 57	α-amylase	BROSI_A0626	37.3%
Dtur_0794	GH 13	α-amylase	AC812_10325	35.3%
Dtur_0852	GH 3	β-glucosidase	M164_2324	58.2%
Dtur_0895	GH 57	α-amylase	TSIB_1115	46.5%
Dtur_0896	GH 57	α-amylase	Calab_2422	40.9%
Dtur_1539	GH 2	β-glucuronidase	Calkro_0120	60.7%
Dtur_1647	GH 10	xylanase	PaelaDRAFT_3013	51.2%
Dtur_1670	GH 36	α-galactosidase	Calla_1244	77.7%
Dtur_1677	GH 4	β-glucosidase	L21TH_1859	47.0%
Dtur_1714	GH 67	α-glucuronidase	Mc24_01903	69.4%
Dtur_1723	GH 3	β-glucosidase	*C. polysaccharolyticus* Xyl3A	46.7%
Dtur_1735	GH 51	β-xylosidase	COB47_1422	70.2%
Dtur_1749	GH 4	α-glucosidase	TRQ7_00895	68.7%
Dtur_1758	GH 38	α-mannosidase	CTN_0786	41.3%
Dtur_1799	GH 1	β-glucosidase	Hore_15280	57.7%
Dtur_1800	GH 43	β-xylosidase	Athe_2555	82.9%
Dtur_1802	GH 2	β-galactosidase	Thewi_0408	42.2%

BLAST analysis was used to determine the closest orthologs of the 66 Dtur CAZymes. Of these 66 enzymes, 56 have their closest orthologs in *D. thermophilum*, with 80–90% amino acid identity. The remaining 10 enzymes have no orthologs in *D. thermophilum*. Seven of the ten unique enzymes in *D. turgidum* are secreted enzymes, including three of the four predicted pectin-degrading enzymes, three of the four predicted xylan-degrading enzymes and the predicted *endo*-arabinase. Only two Dtur enzymes, Dtur_0243 and Dtur_1800, have non-*Dictyoglomus* orthologs with over 80% identity (Tables 2, 3). The nearest non-*Dictyoglomus* orthologs of most of the 66 have <60% identity, showing the uniqueness of the Dtur enzymes. The wide range of organisms these orthologs are found in further demonstrates the uniqueness of this organism. Of the 66 enzymes, 13 have nearest orthologs in *Caldicellulosiruptor* species and 13 have nearest orthologs in *Thermotoga* species. Five orthologs are found in mesophilic *Clostridia* species, four in mesophilic *Mahella* species, and three in thermophilic *Thermoanaerobacter* species. The remaining 28 orthologs are spread over a wide range of mesophilic and thermophilic organisms.

### Degradation of polymeric substrates

#### Substrates reported to be degraded for which genomic and enzymatic support exists

Both *D. turgidum* and *D. thermophilum* are reported to utilize starch, and a number of α-amylases have been cloned and characterized from *D. thermophilum*. The genome of *D. turgidum* codes for three annotated extracellular α-amylases (Dtur_0675; Dtur_0676, and Dtur_1675) as well as four annotated intracellular α-amylases (Dtur_0770; Dtur_0794; Dtur_0895, and Dtur_0896) and six annotated β-glucosidases (Dtur_0157; Dtur_0171; Dtur_0320; Dtur_0384; Dtur_0490, and Dtur_1749). These intracellular enzymes may function in both degradation of starch oligosaccharides transported into the cell as well as degradation of glycogen stored in the cell.

*D. turgidum* is reported to utilize pectin, while no data on pectin utilization was reported for *D. thermophilum*. The genome of *D. turgidum* possesses three annotated secreted pectin lyases (Dtur_0430, Dtur_0431, and Dtur_0432), one secreted pectin esterase (Dtur_0433), an annotated intracellular pectin lyase (Dtur_0435), and an annotated intracellular α-galacturonidase (Dtur_0440).

*D. turgidum* is reported to utilize carboxymethyl cellulose. Because carboxymethyl cellulose is a man-made chemically-modified derivative of cellulose, there are no specific annotated carboxymethyl cellulases present in nature. Unlike cellulose which is crystalline and insoluble in water, carboxymethyl cellulose is an amorphous polymer that is soluble in aqueous solutions. As a result of this solubility, carboxymethyl cellulose is used as a substrate in the assay of a number of enzyme families including xylanases, cellulases, and β-glucanases. The genome of *D. turgidum* has two annotated, secreted xylanases (Dtur_0243 and Dtur_1715) and three secreted annotated cellulases (Dtur_0276; Dtur_0669, and Dtur_1586). The organism also possesses one annotated intracellular xylanase (Dtur_1647), two intracellular cellulases (Dtur_0670 and Dtur_0671) as well as six annotated β-glucosidases (Dtur_0219; Dtur_0289; Dtur_0321; Dtur_0462; Dtur_1723, and Dtur_1799). Assay of the enzymes expressed and purified in this work showed three cellulases (Dtur_0276; Dtur_0670 and Dtur_0671) and two xylanases (Dtur_1647 and Dtur_1715) utilized carboxymethyl cellulose as substrate, producing high levels of reducing sugars from a carboxymethyl cellulose solution (data not shown). These enzymatic assay results confirm the genomic analyses indicating that *D. turgidum* can utilize carboxymethyl cellulose.

#### Substrates reported to be degraded for which genomic and enzymatic support does not exist

*D. turgidum* is reported to utilize crystalline cellulose (Svetlichny and Svetlichnaya, 1988), though the authors report “the organism grew markedly less readily on microcrystalline cellulose, lignin and humic acids.” This in in contrast to *D. thermophilum*, which is reported unable to utilize cellulose. Microbial degradation of crystalline cellulose requires the expression and secretion of multiple cellulases and accessory proteins to decrystallize the cellulose chains and generate soluble, low molecular weight cellodextrins (Brumm, 2013). These cellodextrins are then taken up via membrane transporters and further degraded into glucose monomers in the cytoplasm. Genomic and enzymatic analysis of *D. turgidum* indicate the organism is most likely unable to degrade crystalline cellulose. Comparison of the two genomes indicates *D. turgidum* contains no additional annotated cellulases not found in *D. thermophilum*. All five of the *D. turgidum* cellulases (Dtur_0276; Dtur_0669; Dtur_0670; Dtur_0671 and Dtur_1586) have orthologs in *D. thermophilum* (Dicth_0008; Dicth_0505; Dicth_0506; Dicth_0508 and Dicth_1476, respectively). Analysis of the genome shows a lack of GH9, GH6, GH8, GH12, or GH48 cellulases found in truly cellulytic organisms (Brumm, 2013). Close examination of the genome reveals no cellulases containing CBM2 or CBM3 modules present in cellulose-degrading *Caldicellulosiruptor* species or cellulosomal structures present in cellulose-degrading *C. thermocellum* species within the genome. Assay of the enzymes expressed and purified in this work showed three intracellular enzymes (Dtur_1647; Dtur_0670 and Dtur_0671) produced low levels of reducing sugars from crystalline cellulose (data not shown). The two secreted xylanases (Dtur_0276 and Dtur_1715) produced no reducing sugar from the crystalline cellulose. The lack of activity by the secreted enzymes confirms the genomic analyses indicating that *D. turgidum* most likely cannot utilize crystalline cellulose as a growth substrate. The microcrystalline cellulose preparation used in the original study may have contained glucan, xylan or mannan, resulting in the observed weak growth of the organism on cellulose used in the experiments (Svetlichny and Svetlichnaya, 1988).

The ability of *D. turgidum* to utilize lignin and humic acids is questionable for many of the reasons described above. The authors do not describe the source, purification, and analysis of the lignin and humic acids used in the growth experiments. Depending on the method of purification, lignin is often contaminated with mannan, cellulose and hemicellulose. Humic acids and lignin also contain sugars chemically bonded via ester linkages. Utilization of these sugars may be responsible for the low-level growth seen with these substrates. Thermophilic organisms capable of degrading aliphatic and aromatic organic compounds such as *Geobacillus* species, contain clearly identifiable extended gene clusters with these functions. For example, *Geobacillus* species Y41MC52 possesses three clusters annotated for degradation of aromatic acid molecules, GYMC52_1956 through GYMC52_1962; GYM C52_1990 through GYMC52_2001, and GYMC52_ 3134 through GYMC52_3141. Three similar clusters are found in the related strain *Geobacillus* species Y41MC61. Manual annotation of the *D. turgidum* genome failed to identify orthologs of any of the genes present in the three clusters, confirming that *D. turgidum* cannot utilize the aromatic ring structures found in lignin and humic acids.

#### Substrates not reported to be degraded for which genomic and enzymatic support exists

No data was reported on xylan utilization by *D. turgidum*, however a xylanase has been cloned and expressed from *D. thermophilum*. The genome of *D. turgidum* has two annotated, secreted xylanases (Dtur_0243 and Dtur_1715) and three secreted β-xylosidases (Dtur_1729; Dtur_1739, and Dtur_1740). Genes for intracellular enzymes annotated as feruloyl esterase (Dtur_0242), acetyl xylan esterase (Dtur_0265), xylanase (Dtur_1647), α-glucuronidase (Dtur_1714), and two β-xylosidases (Dtur_1735 and Dtur_1800) may be involved in degradation of oligosaccharides derived from xylan.

Mannans and glucans comprise a diverse group of plant-based polysaccharides that share a β-linked hexose backbone. Among the members of these two groups are mannan, glucomannan, galactomannan, galactoglucomannan, β-glucan, curdlan, and xyloglucan. No data was reported on mannan or glucan utilization by either *D. turgidum* or *D. thermophilum*. The genome of *D. turgidum* codes for two annotated, secreted β-mannanases (Dtur_0097, and Dtur_0277) and one intracellular β-mannanase (Dtur_0629), three secreted annotated cellulases (Dtur_0276; Dtur_0669, and Dtur_1586) and two intracellular cellulases (Dtur_0670 and Dtur_0671), as well as six annotated β-glucosidases (Dtur_0219; Dtur_0289; Dtur_0321; Dtur_0462; Dtur_1723, and Dtur_1799).

Arabinogalactan is a polysaccharide found in many plants, with the highest concentration in larch wood. No data was reported on arabinogalactan utilization by either *D. turgidum* or *D. thermophilum*. The genome of *D. turgidum* codes for a secreted annotated β-galactanase (Dtur_0857) as well as four cytoplasmic β-galactosidases (Dtur_0081; Dtur_0081; Dtur_0505, and Dtur_1802) and one cytoplasmic α-galactosidase (Dtur_1670). Together these enzymes may be adequate for degradation of arabinogalactan as well as galactose-containing oligosaccharides. Annotated genes also code for intracellular fucosidase (Dtur_0315), invertase (Dtur_0551), galacturonidase (Dtur_0440) and β-glucuronidase (Dtur_1539), pectate lyase (Dtur_0435), and chitinase (Dtur_0523).

To verify the activities of some of these enzymes, cloning, expression, and purification was attempted for 30 of the annotated carbohydrase genes. Of these thirty, 16 Dtur enzymes were successfully expressed, purified, and characterized (Table 4). The 16 included two each of GH1 and GH3, four GH5, two GH10, and one each of GH36, GH42, GH43, GH53, GH57, and GH67. The remaining genes either failed to give amplicons of the correct size, or failed to express a soluble protein of the correct molecular weight (Brumm et al., 2011).

**Table 4:** **Enzymatic activity of cloned gene products**.

**Gene**	**GH family**	**Annotated activity**	**Substrates hydrolyzed**
Dtur_0462	GH 1	β-glucosidase	MUA, MUC, MUG, MUX, XG
Dtur_1799	GH 1	β-glucosidase	MUA, MUC, MUG, MUX, XG
Dtur_0852	GH 3	β-glucosidase	MUA, MUC, MUG, MUX
Dtur_1723	GH 3	β-glucosidase	MUA, MUG, MUX
Dtur_0276	GH 5	Cellulase	BG
Dtur_0669	GH 5	Cellulase	BG, GM
Dtur_0670	GH 5	Cellulase	AX, BG, GM, HEC, XG
Dtur_0671	GH 5	Cellulase	GM
Dtur_1647	GH 10	Xylanase	AX, ARA, BG, HEC, MUG, MUX
Dtur_1715	GH 10	Xylanase	AX
Dtur_1670	GH 36	α-galactosidase	XAG
Dtur_0505	GH 42	β-galactosidase	MUA, MUX
Dtur_1729	GH 43	α-arabinase	ARA
Dtur_0857	GH 53	β-galactanase	MUA, XAG
Dtur_0675	GH 57	α-amylase	PUL
Dtur_1714	GH 67	α-glucuronidase	Xylan, xylooligosaccharides

*Legend: MUC, 4-methylumbelliferyl-β-D-cellobioside; MUX, 4-methylumbelliferyl-β-D–xylopyranoside; MUG, 4-methylumbelliferyl-β-D-glucoyranoside; MUA, 4-methylumbelliferyl-α-D-arabinofuranoside; XAG, 5-Bromo-4-chloro-3-indolyl α-D-galactopyranoside; XG, 5-Bromo-4-chloro-3-indolyl β-D-galactopyranoside; AR, AZCL-arabinan; AX, AZCL-arabinoxylan; BG, AZCL-β-glucan; GL, AZCL-galactan; GM, AZCL-galactomannan; HEC, AZCL-hydroxyethyl cellulose; PUL, AZCL-pullulan; XG, AZCL-xyloglucan*.

The two GH1 family members, Dtur_0462 and Dtur_1799, are annotated as β-glucosidases. These two cloned enzymes possess not only the predicted β-glucosidase activity, but also possess β-cellobiosidase, β-galactosidase, β-xylosidase and β-arabinofuranosidase activities (Table 4). GH 3 family members Dtur_0852 and Dtur_1723, also annotated as β-glucosidases, show β-glucosidase, β-xylosidase and β-arabinofuranosidase activity. Dtur_0852 also possesses β-cellobiosidase activity, which is absent in Dtur_1723 (Table 4).

The four GH5 family annotated cellulases show a wide range of activities. Three of the GH5 family members hydrolyze a limited number of substrates. Dtur_0276 possesses only β-glucanase activity, while Dtur_0671 possesses only β-mannanase activity. Dtur_0669 possesses both β-mannanase and β-glucanase activities. None of the three possess β-glucosidase, β-cellobiosidase, β-galactosidase, or β-xylosidase activity. In contrast to these three enzymes, Dtur_0670 (Dtur CelA) possesses both endo-activity and exo-activity on a wide range of substrates (Table 4). Dtur_0670 possesses endoglucanase activity on a number of insoluble chromogenic substrates including AZCL-HE cellulose, AZCL-β-glucan, and AZCL-xyloglucan, endomannanase activity on AZCL-glucomannan, endoxylanase activity on AZCL-arabinoxylan, as well as β-glucosidase and β-cellobiosidase activity (Brumm et al., 2011). None of the GH5 family members released physiologically relevant amounts of sugar from crystalline cellulose even under prolonged incubation.

The two GH10 family xylanases show significantly different activities. Dtur_1715 possesses only endoxylanase activity, with no other detectable *endo*- or *exo*-activities. Dtur_1647 (XynA) displays *endo*-activity on β-(1,4)-linked pentose substrates such as xylan, arabinoxylan, and linear arabinan and β-(1,4)-linked hexose substrates such as β-glucan and hydroxyethyl cellulose. XynA also possesses β-glucosidase, β-xylosidase and β-cellobiosidase activity (Table 4).

The GH42 family member, Dtur_0857 predicted to be a β-galactanase, possesses no *endo*-activity, but instead possesses β-galactosidase and β-arabinofuranosidase activities. The GH43 family member, Dtur_1729, does not possess β-xylosidase and β-arabinofuranosidase activity as expected from the annotation, but instead possesses only *endo*-β-arabinase activity. The GH57 family member, Dtur_0675, possesses α-amylase activity as predicted. The GH67 family member, Dtur_1714, shows strong α-glucuronidase activity on xylan and xylan oligosaccharides as predicted (Gao et al., 2011). Comparison to structural orthologs indicate that all 16 enzymes possess a single active site, with the differences in substrate range being a function of active site accessibility for each enzyme. There is no evidence of multiple active sites in any of the enzymes examined. The properties of these 16 enzymes show *D. turgidum* possesses the enzymes capable of hydrolyzing arabinoxylan, arabinan and arabinogalactan, β-glucan, mannan, and glucomannan to usable sugars. Failure to obtain active pectinase clones prevented us from confirming the ability of the organism to utilize pectin.

### Energy generation

Dtur is predicted to utilize the Embden–Meyerhof–Parnas pathway to produce ATP, reducing equivalents, and fermentation products from monosaccharides. Predicted products from pyruvate include lactate (Dtur_0700), acetate via acetyl-CoenzymeA (acetyl-CoA) (Dtur_0260; Dtur_0261, and Dtur_0262), and ethanol via acetyl CoA (Dtur_0260; Dtur_0261, and Dtur_0262) and acetaldehyde (Dtur_0484 and Dtur_1632). Hydrogen production is predicted by the presence of three hydrogenase gene clusters. The annotation reveals a partial *hypA* operon (Dtur_0074 through Dtur_0080) upstream of a hydrogenase assembly cluster (Dtur_0086 through Dtur_0090). Additional hydrogenase genes are located in a downstream cluster (Dtur_0556 through Dtur_0561). These metabolic predictions are in agreement with the published microbiological studies (Svetlichny and Svetlichnaya, 1988) showing the organism produces acetate, lactate, ethanol, CO_2_, and H_2_ during fermentation on sugars.

Based on the genome annotation, Dtur possesses an incomplete, reductive TCA cycle. This cycle allows the organism to convert acetate to pyruvate, oxaloacetate, and eventually to α-ketoglutarate. The α-ketoglutarate generated in this pathway can then be utilized for production of glutamate and other amino acids. Based on the annotation, the organism is unable to synthesize citrate from oxaloacetate and acetyl CoA.

Dtur has an extremely simple respiratory system. The genome codes for no respiratory cytochromes. The pathways for production of aminolevulinic acid from either glycine and succinyl-CoA or glutamate are both absent in Dtur. No tetrapyrroles (hemes, sirohemes, or corrinoids) are synthesized by Dtur, and the organism has no ABC-type heme transporters to utilize exogenous heme. The organism is also lacking the pathway for ubiquinone biosynthesis, indicating either ubiquinone is scavenged from the environment, or an alternate electron acceptor is utilized, like ferridoxin (Dtur_0730) or ferredoxin-like proteins (Dtur_0076; Dtur_0457; Dtur_0556; Dtur_0730; Dtur_0774, and Dtur_1717). The proton gradient needed for ATP generation is produced by NADH oxidoreductase (Dtur_0558; Dtur_0559; Dtur_0916; Dtur_0919, and Dtur_1091), and succinate dehydrogenase (Dtur_0445). ATP is generated by proton translocation via an F0F1-type ATP synthase (Dtur_129 through Dtur_135). *D*. turgidum also possesses a V-type ATP synthase (Dtur_1499 through Dtur_1506), the function of which is unclear. The V-type ATP synthase may also be used for ATP generation, or may it may hydrolyze ATP to generate proton or ion gradients for transport.

### Other metabolic pathways

As expected from its small genome size, *D. turgidum* does not possess a full set of biosynthetic and metabolic capabilities. *D. turgidum* appears able to synthesize all 20 amino acids from carbohydrate precursors, but as mentioned previously, is unable to metabolize the majority back to carbohydrates. Like the amino acid situation, *D. turgidum* is able to synthesize fatty acids, but not degrade them. The organism appears to synthesize folate, pyridoxal 5'-phosphate, thiamine, NAD from aspartate, and ascorbate from glucose or galactose, but is lacking pathways for biosynthesis of biotin, pantothenate or flavins.

Carbon monoxide is utilized by strict anaerobes via the Wood-Ljungdahl pathway (Techtmann et al., 2009), using the anaerobic CO dehydrogenase/acetyl CoA synthase complex. This complex catalyzes the complex multistep anaerobic reactions that include oxidizing CO to CO_2_, formation of H_2_, and biosynthesis of acetyl CoA. This pathway is found in thermophilic anaerobes such as *T. tengcongensis* and *M. thermoacetica* as well as in two *G. thermoglucosidasius* species. Manual curation of the genome indicates that D. turgidum does not possess the Wood-Ljungdahl pathway and is unable to utilize carbon monoxide as a carbon and energy source.

### DNA replication, recombination, and repair

IMG (DOE JGI) annotation methods identified 61 COG functional category L members (Table 1) for *D. turdigm*. Manual reannotation of each L category gene uncovered six mis-annotated genes. Five genes annotated as excinuclease ATPase subunits (Dtur_0247, 1011, 1053, 1153, 1667) are more likely ABC type transporters and an endonuclease (Dtur_0036) has supporting evidence to be annotated as a xylose isomerase. These genes were removed from the compilation shown in Table 5. A complete review of the annotated genes for *D. turdigum* identified a number of missed and overlooked genes that properly belong in the L COG family which totals 85 members in our revised tabulation of the genome (Table 5). Even though the 16S rRNA genes of the only two described species of *Dictyoglomus, D. turgidum* (CP001251) and *D. thermophilum* (CP001146), share 99% sequence identity, the divergence of their orthologous replication proteins is significant, as described below.

**Table 5:** *****D. turdigum*** annotated DNA replication, recombination, and repair enzymes**.

**Dtur gene**	**Annotation**	**Nearest neighbor/% identity**
0745	ATP-dependent DNA helicase	*Thermoanaerobaculum aquaticum*/43
1397	ATP-dependent DNA helicase PcrA	*Caloranaerobacter azorensis*/49
1140	ATP-dependent DNA helicase RecG	*Halothermothrix orenii*/52
0780	ATP-dependent DNA ligase	uncultured *Acidobacteria*/56
1514	Bacterial nucleoid DNA-binding protein	*Symbiobacterium thermophilum*/66
1151	Cell division protein FtsK	*Thermoanaerobacterium aotearoense*/41
0001	Chromosomal replication initiator protein DnaA	*Pelotomaculum thermopropioni*/59
1468	Chromosome segregation and condensation protein ScpA	*Caldisalinibacter Kiritimati*/28
1467	Chromosome segregation and condensation protein ScpB	*Dethiobacter alkaliphilus*/45
0947	Chromosome segregation protein SMC	*Caloranaerobacter azorensis*/27
1104	Competence protein ComE	*Clostridium purinilyticum*/29
1103	Competence protein ComEA	*Clostridium* sp. CAG:273/55
1614	Crossover junction endodeoxyribonuclease RuvC	*Alkaliphilus oremlandii*/50
0771	Deoxyinosine 3'endonuclease (endonuclease V)	bacterium JGI-24/54
1268	DNA and RNA Helicase	candidate division *Zixibacteria*/57
1547	DNA gyrase B subunit	*Thermoanaerobacter* sp. YS13/66
1263	DNA gyrase, A subunit	*Mahella australiensis*/54
1478	DNA integrity scanning protein DisA	*Bacillus* sp. CMAA 1185/52
1078	DNA mismatch repair protein MutL	*Caldanaerobacter subterraneus*/42
1077	DNA mismatch repair protein MutS	*Clostridium thermocellum*/46
0884	DNA or RNA helicase of superfamily II (UvrB)	*Caldanaerobacter subterraneus*/68
0104	DNA polymerase beta domain-containing protein	*Syntrophaceticus schinkii*/57
0317	DNA polymerase beta domain-containing protein	marine sediment metagenome/38
0545	DNA polymerase beta domain-containing protein	*Microgenomates* bacterium/39
1295	DNA polymerase beta domain-containing protein^*^	*Chloracidobacterium thermophilum*/60
0882	DNA polymerase I 3'-5' exonuclease & polymerase domains	*Tepidanaerobacter acetatoxydans*/44
1391	DNA polymerase III alpha subunit	*Caloranaerobacter* sp. TR13/52
1105	DNA polymerase III delta subunit	*Alkaliphilus oremlandii*/29
0257	DNA polymerase III gamma/tau subunits	*Thermincola potens*/41
0789	DNA polymerase III gamma/tau subunits	marine sediment metagenome/39
1551	DNA polymerase III sliding clamp subunit beta	*Clostridium acidurici*/37
1600	DNA Polymerase X	*Caldisericum exile*/55
1316	DNA primase N	*Lachnospiraceae* bacterium/38
1527	DNA protecting protein DprA	*Caldanaerobacter subterraneus*/43
1549	DNA recombination protein RecF	*Clostridium aceticum*/36
1625	DNA repair exonuclease	*Thermobaculum terrenum*/33
0327	DNA repair photolyase	*Parcubacteria* bacterium/41
0463	DNA repair photolyase	*Caldicellulosiruptor owensensis*/66
1479	DNA repair protein RadA	*Thermoanaerobacterium xylanolyticum*/48
0881	DNA repair protein RADC	*Paenibacillus* sp./50
1047	DNA repair protein RecN	*Caloramator australicus*/42
1321	DNA repair protein RecO	*Microgenomates* bacterium/29
0015	DNA replication and repair protein RecF	*Fervidobacterium nodosum*/80
1526	DNA topoisomerase type I	*Carboxydothermus hydrogenoformans*/54
1522	Double-stranded DNA repair protein Rad50	*Thermoanaerobacter siderophilus*/26
1626	Double-stranded DNA repair protein Rad50	*Thermofilum* sp./25
0264	Endonuclease IV	*Thermincola potens*/36
1485	Excinuclease ABC subunit C	*Acetohalobium arabaticum*/45
0885	Excinuclease ATPase subunit (ABC-ATPase UvrA)	*Thermosediminibacter oceani*/64
1613	Holliday junction DNA helicase RuvA	*Thermodesulfovibrio yellowstonii*/34
1612	Holliday junction DNA helicase RuvB	*Caldicellulosiruptor obsidiansis*/54
0792	Holliday junction resolvasome helicase subunit	*Carboxydothermus hydrogenoformans*/54
1284	Integrase^*^	*Acetothermus autotrophicum*/35
0886	Methylated DNA-protein cysteine methyltransferase	*Anaerococcus lactolyticus*/40
1227	Mg-dependent Dnase—deoxyribonuclease TatD	*Bacillus* sp. SA1-12/47
1308	Mismatch repair ATPase (MutS family)	*Peptococcaceae* bacterium/40
1294	Modification methylase, type III R/M system^*^	*Sulfurihydrogenibium yellowstonense*/65
0341	N6-adenine-specific methylase	*Caloranaerobacter azorensis*/65
1141	N6-adenine-specific methylase	*Thermoanaerobacter ethanolicus*/44
1497	Poly(A) polymerase	*Peptococcaceae* bacterium/36
0683	Predicted EndoIII-related endonuclease	*Thermodesulfatator indicus*/58
0846	Predicted EndoIII-related endonuclease	*Candidatus Methanoperedens* sp./32
1024	Predicted endonuclease involved in recombination	*Veillonella atypica*/44
1530	Predicted endonuclease related to Holliday junction resolvase	*Caldicellulosiruptor saccharolyticus*/42
1709	Predicted exonuclease	marine sediment metagenome/61
0102	Predicted nucleic acid-binding protein contains PIN domain	*Leptospira wolbachii*/47
1441	Primosomal protein N' (replication factor Y)	human gut metagenome/29
1507	Putative chromosome partioning protein	*Plasmodium knowlesi*/38
1162	RecA/RadA recombinase	*Thermosediminibacter oceani*/73
1524	Recombinase XerD	Symbiobacterium thermophilum/47
0259	Recombination protein RecR	*Oxobacter pfennigii*/58
1358	Replicative DNA helicase	*Acetohalobium arabaticum*/56
0014	Reverse gyrase	*Fervidobacterium nodosum*/79
0708	Ribonuclease HII	*Kosmotoga pacifica*/35
1531	Ribonuclease HII	*Clostridium* sp./59
0118	Single stranded nucleic acid binding protein (R3H domain)	*Butyrivibrio* sp./45
1602	Single-stranded DNA exonuclease RecJ	*Clostridium thermocellum*/36
1362	Single-stranded DNA-binding protein	*Syntrophothermus lipocalidus*/52
0786	Single-stranded nucleic acid binding protein (R3H domain)	*Syntrophobacter fumaroxidans*/59
0880	Smc1 chromosome segregation protein, putative	marine sediment metagenome/28
0202	Thermostable 8-oxoguanine DNA glycosylase	*Thermotoga maritima*/59
1511	Transcription-repair coupling factor	marine sediment metagenome/40
1297	Type III restriction endonuclease subunit R^*^	bacterium JGI-6/67
1473	Tyrosine recombinase XerD	*Tepidanaerobacter acetatoxydans*/51
1393	Uracil-DNA glycosylase	*Nitrospira moscoviensis*/54

Prophage related genes are marked^*^.

The genome of *D. turdigum* possesses 85 annotated genes for DNA replication, recombination and repair (Table 5), 78 of which have their closest ortholog to Dicth genes, whereas 6 have no orthologs in Dicth (Dtur_0102, 0317, 0545, 1284, 1294, 1297). The last four genes are part of a prophage that is not present in *D. thermophilum*. There are two genes in this category that are present in *D. thermophilum* but not *turdigum*, including a deoxyribodipyrimidine photolyase (Dicth_0072) and a DNA modification methylase (Dicth_0253). Only one gene (Dtur_1514), annotated as a bacterial nucleoid DNA-binding protein has 100% identity to its Dicth ortholog. On average the genes in this COG category share 85% identity with their Dicth counterparts, with the lowest at 66% identity (Dtur_1626, double-stranded DNA repair protein Rad50). Based on blastP analysis the most striking feature of this class of genes is how dissimilar they are to other sequenced genes and genomes in the database, other than *D. thermophilum*. This is apparent when the next nearest neighbors to *D. turdigum* DNA replication proteins are tabulated (Table 5). On average the genes in this COG category share 48% amino acid identity to nearest neighbor genes (25–80% range) and the cross section of homology is widespread among taxa that are primarily anaerobic, thermophilic, or halophilic. Only two of the genes share homology to Thermotogae (Dtur_0202 and 0708) and 3 to *Caldicellulosiruptor* (Dtur_0463, 1530, and 1612). The greatest frequency of nearest neighbor orthologs after *D. thermophilum* are to clostridial (9%) and *Thermoanerobacterium* (8%) genus members, followed by unknown metagenomic genes (6%). The low homology of *Dictyoglomus* replication proteins to orthologs in other organisms is another testament to how phylogenetical unique the genus is.

In spite of its preferred growth temperature of 72°C (Svetlichny and Svetlichnaya, 1988), *D. turdigum* has an extremely low (33.96 mol%) G+C content, which seems counterintuitive to genome stability and repair (Ishino and Narumi, 2015). Dtur does possess a reverse gyrase (Dtur_0014), a hallmark enzyme that is systematically present in all hyperthermophiles (Brochier-Armanet and Forterre, 2007), which introduces positive supercoils in DNA and thereby protects it from unwinding. Dtur and Dicth do not appear to contain genes for exonuclease III, a DNA-repair enzyme that hydrolyzes the phosphodiester bond 5′ to an abasic site in DNA, which is commonly induced by heat. However, they both possess endonuclease IV, which has been shown to perform a similar abasic site processing function in *Thermotoga maritima* (Haas et al., 1999).

**Figure 4:**
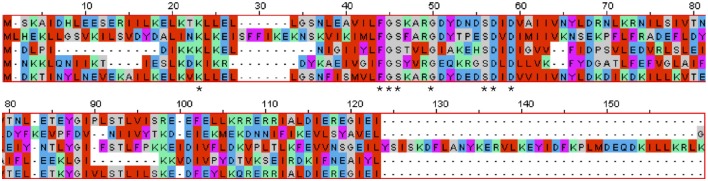
**Multiple sequence alignment of all five ***Dictyoglomus*** (4 from ***turgidum*** and one from ***thermophilum***) nucleotidyltransferase domains of DNA polymerase beta family protein sequences using MAFFT software (Katoh et al., 2002)**. From top to bottom: Dtur_0104, 114AA; Dtur_0317, 121AA, Dtur_0545, 135AA, Dtur_1295, 99AA, Dicth_0227, 114AA. Dtur_1295 is located in the prophage region of *D. turdigum*.

*D. turgidum* possesses 7 DNA replication and repair genes annotated to contain a nucleotidyltransferase (NT) domain, a superfamily that includes DNA polymerase beta domain-containing proteins (NT_Pol-beta), family X and poly-A DNA polymerases, as well as other proteins (Aravind and Koonin, 1999). The majority of the NTs are characterized by a distinct amino acid residue pattern, namely hG[GS]x(9,13)Dh[DE]h (x indicates any amino acid and h indicates a hydrophobic amino acid) that are essential for catalysis, which is true for all 7 NT domain containing genes in *D. turdigum*. Three of the *D. turdigum* NT domain-containing DNA repair proteins are larger than 450 amino acids (Dtur_0257, 1497, and 1600), whereas four members further annotated as belonging to the Pol-beta subfamily only encode 99–135 amino acids (Figure 4). DNA polymerase B is a proofreading-proficient enzyme thought to be involved with DNA repair activities in eubacteria (Wijffels et al., 2005) and replication in archaea (Kelman and Kelman, 2014), however there are no known thermophilic bacterial DNA polymerase B genes. A new, ancestral family of polB type nucleotidyltransferases designated as MNT (minimal nucleotidyltransferases) has been described (Aravind and Koonin, 1999) that are one-half to one-third the size of the larger orthologs. They are not uncommon as 258 cases can be found in the protein NCBI database “dna polymerase beta domain-containing protein” in 129 different microbes as of January 2016. However, there are no known biochemical studies showing whether these diminutive NTs are catalytically functional monomeric enzymes or whether they are part of a larger multimeric complex. The four NT_Pol-beta genes found in *D. turdigum*, one of which is associated with the prophage element discussed below, is an unsolved mystery as to the function these diminutive proteins might play, particularly with regard to the lack of a PolB enzyme in this hyperthermophile.

### Functional analysis of *D. turdigum* DNA polymerase I

*D. turgidum* possesses four sets of DNA polymerases: Pol X (Dtur_1600), Poly(A) polymerase (Dtur_1497), DNA polymerase I (Dtur_0882), and a minimal DNA polymerase III set of subunits (alpha, Dtur_1391; beta, Dtur_1551; delta, Dtur_1105, and gamma/tau, Dtur_0257 and _0789). In a survey looking for thermostable reverse transcriptase (RT) activity, the DNA polymerase I (PolI) from *Dictyoglomus thermophilum* strain Rt46B.1 has been cloned and expressed (Shandilya et al., 2004). While the enzyme did not exhibit RT activity, it did show significant thermal stability at 85°C compared to eight other enzymes being studied. Presumably its ortholog behaves the same, as Dicth_0729 shares 90% identity (770/856) and 96% positives (826/856) at the amino acid level with Dtur_0882.

**Figure 5:**
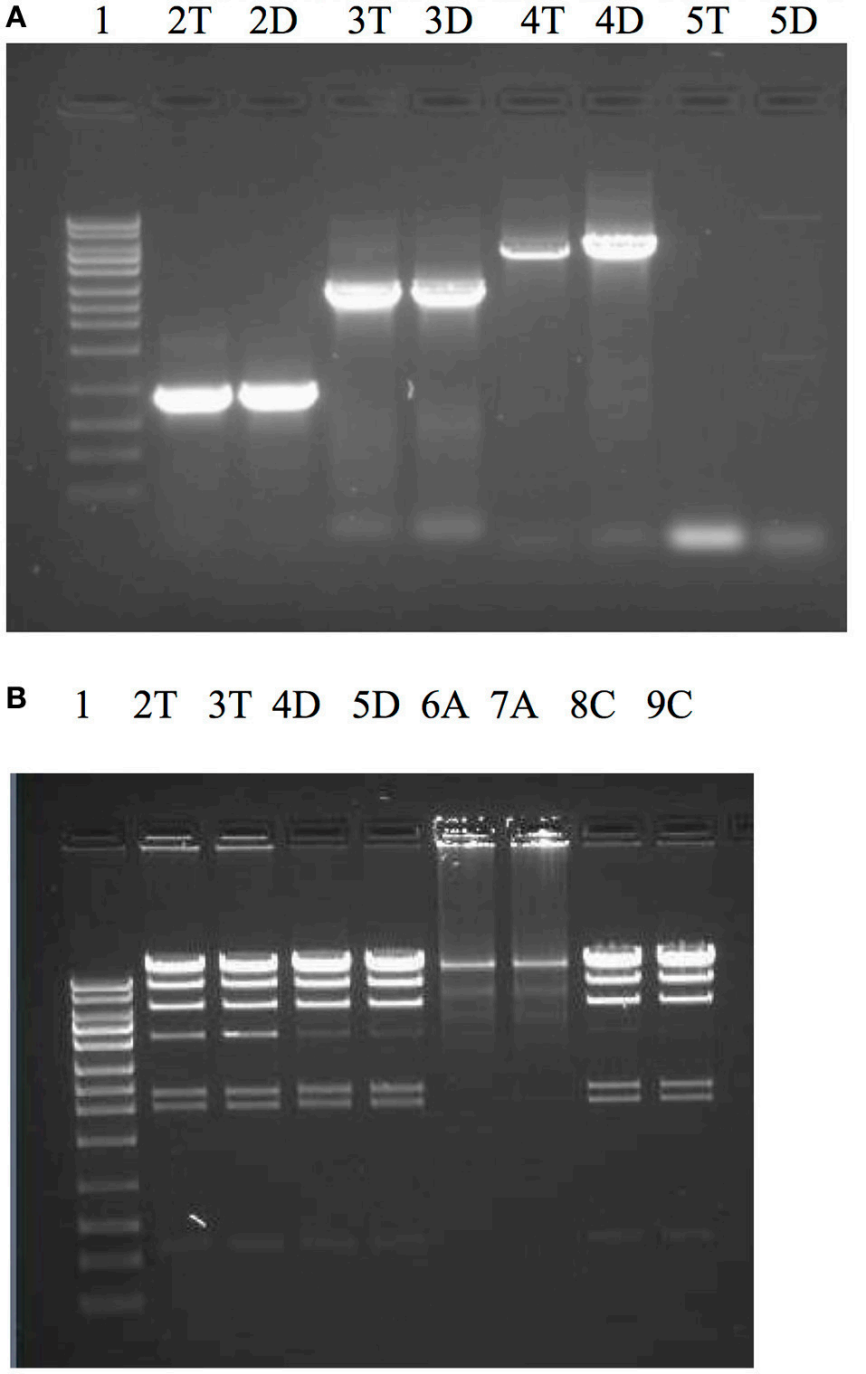
**Comparison of PCR efficacy between Dtur_0882 and Taq DNAP (A)** and 3′-5′ exonuclease activity between Dtur_0882; Taq and OmniAmp DNAP **(B)**. PCR amplicons of 0.9 kb (lane A2), 2.8 kb (lane A3), 5 kb (lane A4), or 10 kb (lane A5) were produced by Taq (T) or Dtur (D) DNAP. To assess exonuclease activity (lanes B2-9) lambda DNA restriction digested with Hind III was incubated with 5U of Taq (T), Dtur (D) or OmniAmp (A) DNAP (in duplicate) overnight at 37°C in PCR buffer. Lane 1 is a 1 kb DNA ladder (Promega).

Dtur_0882 is a PolA type polymerase annotated to contain a 5′-3′ and 3′-5′ exonuclease domain in addition to the DNA-directed DNA polymerase domain. With the except of *Rhodothermus marinus* PolA (PMID:11483153) and the enzyme OmniAmp polymerase (Chander et al., 2014) every thermostable PolA enzyme characterized thus far lacks a functional 3′-5′ exonuclease domain associated with proofreading and enzyme fidelity. Because the high temperature growth conditions for *D. turdigum* are very similar to *Thermus aquaticus* (Taq), but the amino acid identities are so different between the two PolI enzymes (41%, 351/847), the utility of Dtur_0882 as a PCR enzyme was evaluated and compared with Taq DNAP (Figure 5). The 3′-5′ exonuclease activity of both enzymes were also compared with the thermostable proof reading PolA enzyme called OmniAmp polymerase (Chander et al., 2014). Dtur_0882 produced the same yield of amplicon for the 0.9 and 2.8 kb reactions as Taq DNAP (Figure 5A lanes 2, 3), but was more efficient at amplifying the 5 and 10 kb primer templates (Figure 5A lanes 4, 5). As with Taq DNAP, Dtur_0882 does not appear to have any measurable 3′-5′ exonuclease activity (Figure 5B lanes 2/3 compared to 4/5) as opposed to a strong exonuclease activity demonstrated by the proofreader OmniAmp DNAP (Figure 5 lanes 6/7).

### Prophage and CRISPR elements

*D. turgidum* possesses two regions containing CRISPR repeats, suggesting the previous exposure to phage(s). The first CRISPR region, located between 470870 and 474424 nucleotides in the genome codes for 54 repeats. The repeat sequence is 30 nucleotides long, and the spacer average length is 36 nucleotides long. The second CRISPR region, located between nucleotides 6151530 and 617311 in the genome codes for 33 repeats. The repeat sequence is the same as the first CRISPR region, and the spacer average length is 36 nucleotides long. No CRISPR-associated proteins are in the vicinity of the first CRISPR region. Upstream of the second CRISPR region are eight CRISPR-associated proteins (Table 5), while downstream of the second CRISPR region is a four-gene insert coding for biosynthetic enzymes (Dtur_0614—Dtur_0617) and eight additional CRISPR-associated proteins. *D. thermophilum* shows a similar organization of its CRISPR-associated proteins (Table 6), with a larger, 15-gene insert coding for biosynthetic enzymes (not shown).

**Table 6:** *****D. turgidum*** CRISPR-associated proteins**.

**Gene**	**Annotation**	**Orthologs**
Dtur_0606	CRISPR-associated protein, TM1812 family	Dicth_0458
Dtur_0607	CRISPR-associated protein DxTHG motif protein	Dicth_0187
Dtur_0608	CRISPR-associated RAMP protein, Csm5 family	Dicth_0186
Dtur_0609	CRISPR-associated RAMP protein, Csm4 family	Dicth_0185
Dtur_0610	CRISPR-associated RAMP protein, Csm3 family	Dicth_0184
Dtur_0611	CRISPR-associated RAMP protein, Csm2 family	Dicth_0183
Dtur_0612	CRISPR-associated RAMP protein, Csm1 family	Dicth_0182
Dtur_0613	CRISPR-associated protein, Csx3 family	Dicth_0181
Dtur_0618	CRISPR-associated protein, Cas2 family	Dicth_0165
Dtur_0619	CRISPR-associated protein, Cas1 family	Dicth_0164
Dtur_0620	CRISPR-associated endonuclease, Cas4 family	Dicth_0163
Dtur_0621	CRISPR-associated helicase, Cas3 family	Dicth_0162
Dtur_0622	CRISPR-associated protein, Cas5h family	Dicth_0161
Dtur_0623	CRISPR-associated protein, Csh2 family	Dicth_0160
Dtur_0624	CRISPR-associated protein, Csh1 family	Dicth_0159
Dtur_0625	CRISPR-associated protein, Cas6 family	Dicth_0158

An incomplete prophage sequence was identified by PHAST (Hubisz et al., 2011; Zhou et al., 2011) as a 17,407 base insert from 1,301,419 to 1,318,825 (Dtur_1284-1300) and confirmed as foreign DNA by IslandViewer Software (Hsiao et al., 2003) (1,298,422 to 1,317,048 containing genes Dtur_1282 through Dtur_1297). The prophage contains an integrase (Dtur_1284) followed by four annotated putative lipoproteins that potentially form part of a *beta*-barrel assembly machinery (Dtur_1285; Dtur_1286; Dtur_1288, and Dtur_1289) and a Type III restriction system (Dtur_1294 and Dtur_1297). The four annotated lipoprotein genes are closely related, as they share 79–88% amino acid identity. Inspection of the amino acid sequence reveals a unique periodicity of aspartic (D) and glutamic (E) residues to hydrophobic residues. The reason for this novel periodicity is due to the seven back to back, nearly perfect 49–52 amino acid tandem repeats found in these proteins (data not shown). Tandem repeat proteins are ubiquitous (Jernigan and Bordenstein, 2015), but the unique signature sequence found here is only partially common to a handful of hypothetical proteins found in bacteria. Additional work is needed to clarify the function of these four repeated proteins with seven tandem internal repeats. This genomic island is unique to *D. turgidum*. *D. thermophilum* possesses an ortholog to only one of the four lipoproteins and no Type III restriction system proteins.

### Thermophily, stress responses, and heat shock proteins

**Figure 6:**
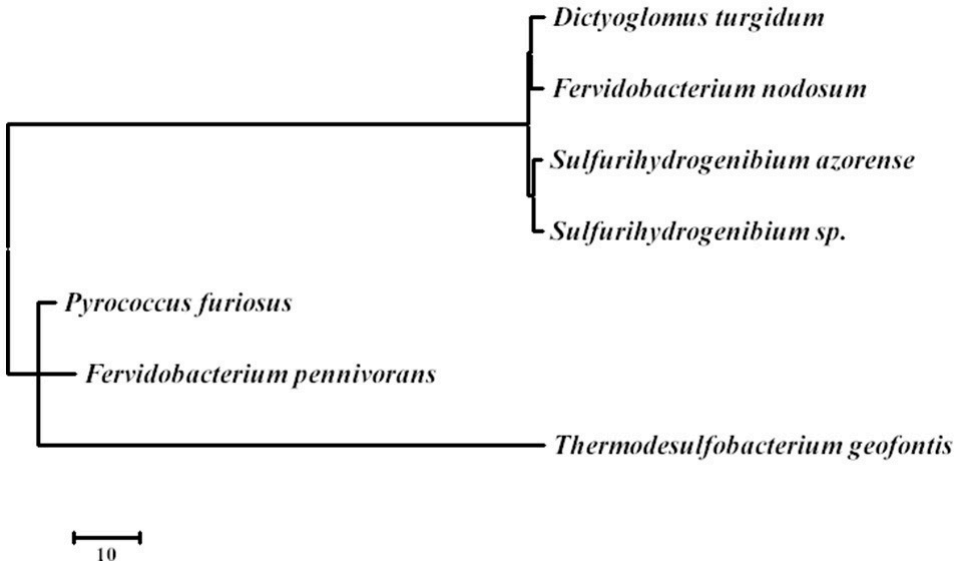
**Evolutionary relationships of reverse gyrase**. The evolutionary history was inferred by using the Maximum Likelihood method based on the JTT matrix-based model (Tamura et al., 2011) The tree with the highest log likelihood (−22290.6302) is shown. Initial tree(s) for the heuristic search were obtained automatically by applying Neighbor-Join and BioNJ algorithms to a matrix of pairwise distances estimated using a JTT model, and then selecting the topology with superior log likelihood value. The tree is drawn to scale, with branch lengths measured in the number of substitutions per site. The analysis involved 7 amino acid sequences. All positions containing gaps and missing data were eliminated. There were a total of 1122 positions in the final dataset. Evolutionary analyses were conducted in MEGA7 (Kumar et al., 2016). UniPpot sequences used for the analysis were: B8DYH3, *Dictyoglomus turgidum* strain DSM 6724; A7HMS7, *Fervidobacterium nodosum* strain DSM 5306; H9UDK4, *Fervidobacterium pennivorans* strain DSM 9078; C1DT23, *Sulfurihydrogenibium azorense* strain DSM 15241; B2V6S9, *Sulfurihydrogenibium* sp. strain YO3AOP; F8C2X1, *Thermodesulfobacterium geofonti*s strain OPF15, and P95479; *Pyrococcus furiosus* strain DSM 3638.

*D. turgidum* in common with *D. thermophilum* has a complement of heat shock proteins typical of thermophilic bacteria, including a single GroEL/ES locus encoding Hsp60 and a DnaK/DnaJ locus encoding Hsp70 and cochaperones GrpE. Interestingly, in both *D turgidum* and *D. thermophilum*, the reverse gyrase is encoded in a gene cluster shared with recJ and revG, closely linked to the DnaK/DnaJ operon. Heat shock regulation is enigmatic since the genome lacks both CIRCE elements and sigma32 SOS regulation. It is tempting to speculate that conditional expression of the reverse gyrase under high temperature growth conditions might be a mechanism for regulating DNA positive supercoiling in concert with the heat shock response. The phylogeny of the reverse gyrase is extraordinary. The reverse gyrase is most closely related to orthologs in *Fervidobacterium* species as shown in Figure 6. This phylogenetic position of the reverse gyrase does not conform to the 16S rRNA phylogeny (Figure 3) where *D. turgidum* is most closely related to *Caldicellulosiruptor* species. This suggests that lateral gene transfer of the reverse gyrase may have taken place.

### Morphological characteristics

Microbiological testing of *D. turgidum* indicate the organism stains Gram-negative (Svetlichny and Svetlichnaya, 1988). The *D. turgidum* genome contains a cluster of 10 genes potentially coding for outer membrane proteins characteristic of Gram-negative organisms and outer membrane lipid biosynthesis (Dtur_0814 through Dtur_0824). This cluster contains genes coding for a TamB (Dtur_0814), two BamA orthologs (Dtur_0815 and Dtur_0816), and two outer membrane chaperone Skp (OmpH) orthologs (Dtur_0817 and Dtur_0818), all potentially involved in outer membrane transport and assembly. This cluster of genes provides genomic support for the observed Gram-negative membrane structure observed in electron micrographs (Svetlichny and Svetlichnaya, 1988). Following these five genes, the *D. turgidum* genome contains a lipid biosynthesis cluster coding for the first four enzymes of Lipid A biosynthesis, LpxD (Dtur_0819), LpxC (Dtur_0820), LpxA (Dtur_0821), and (Dtur_0823) and an ortholog of FabZ (beta-hydroxyacyl-(acyl-carrier-protein) dehydratase, Dtur_0821). The final gene in the cluster (Dtur_0824) is a hypothetical protein related to LpxB. This 10-gene cluster has the identical organization in *D. thermophilum*, with individual genes averaging 80% identity to its Dtur ortholog. The cluster appears to be unique to *Dictyoglomus* species, as no similar cluster is found in any other sequenced organisms. The individual genes also show little homology to orthologs in other species, with observed amino acid identities being ≤30% for all genes in the cluster.

Downstream of this ten-gene cluster is an annotated cluster of six proteins potentially involved in Gram-negative outer membrane efflux including orthologs of an ABC transporter ATP-binding protein (Dtur_0835), two of TolC (Dtur_0836 and Dtur_0837), HlyD (Dtur_0838) an ABC transporter permease protein (Dtur_0839) and a predicted transmembrane protein (Dtur_0840). An identical cluster is found in *D. thermophilum* (DICTH_0678 through DICTH_0683). A similar cluster is found in *Meiothermus taiwanensis* DSM 14542 as well as other *Meiothermus* and *Thermus* species. No orthologs of this cluster are found in any sequenced Thermotogales or Firmicutes species.

**Figure 7:**
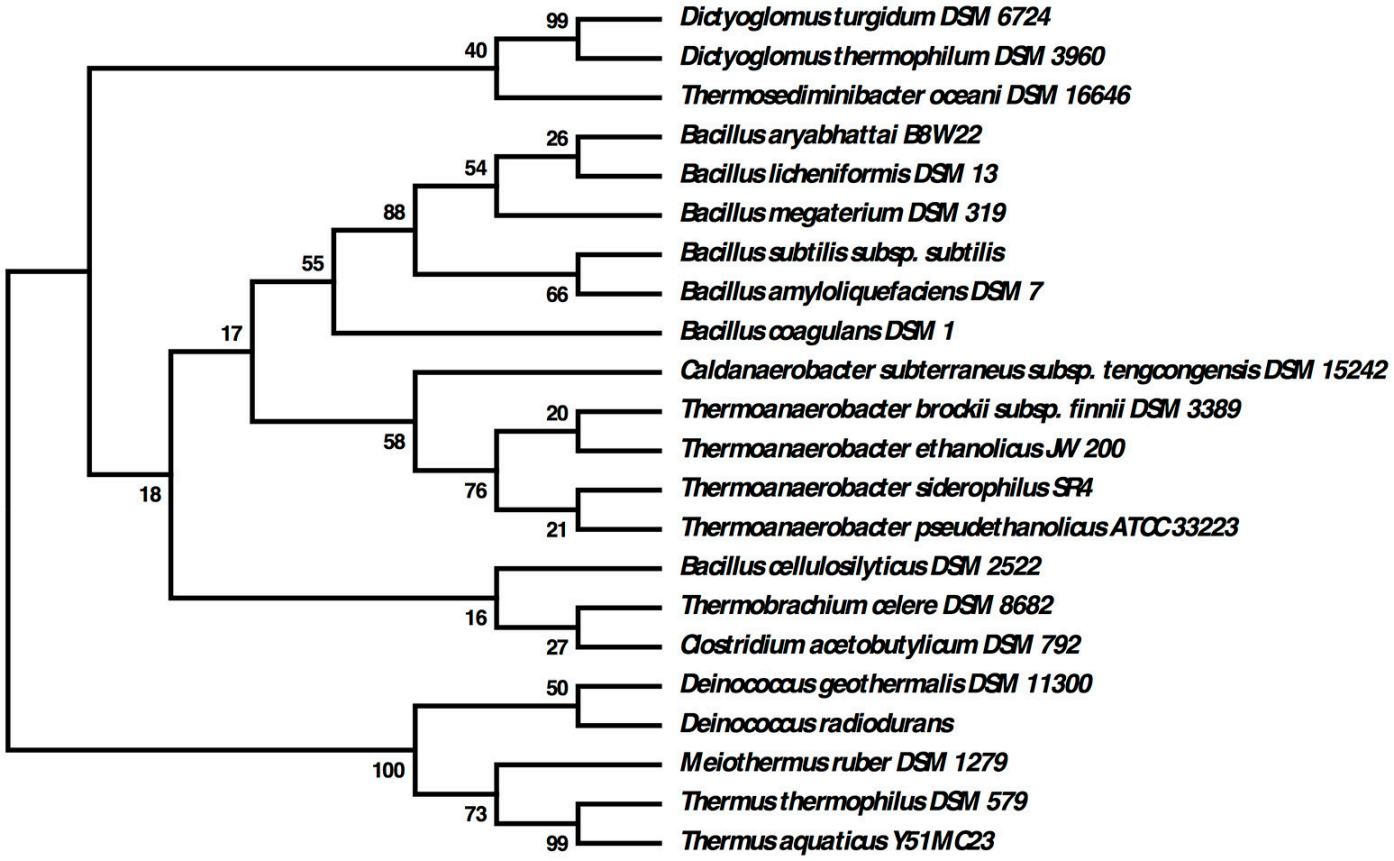
**Evolutionary relationships of SpoVS proteins**. The evolutionary history was inferred by using the Maximum Likelihood method based on the JTT matrix-based model. The bootstrap consensus tree inferred from 550 replicates is taken to represent the evolutionary history of the taxa analyzed. Branches corresponding to partitions reproduced in less than 50% bootstrap replicates are collapsed. The percentage of replicate trees in which the associated taxa clustered together in the bootstrap test (550 replicates) are shown next to the branches. Initial tree(s) for the heuristic search were obtained automatically by applying Neighbor-Join and BioNJ algorithms to a matrix of pairwise distances estimated using a JTT model, and then selecting the topology with superior log likelihood value. The analysis involved 22 amino acid sequences. All positions containing gaps and missing data were eliminated. There were a total of 86 positions in the final dataset.

Many thermophilic bacteria possess complex morphologies, with varying shapes seen under different growth conditions. Examples of this include “rotund bodies” in *Thermus aquaticus* (Brumm P. J. et al., 2015), the outer membrane “toga” of *Thermotoga maritima* (Huber et al., 1986) and the multicellular spheres of *D. turgidum* (Svetlichny and Svetlichnaya, 1988). Regulation of these morphologies may be controlled by the action of SpoVS (Brumm P. J. et al., 2015). *D. turgidum* possesses a gene coding for SpoVS (Rigden and Galperin, 2008) (Dtur_0800) similar to SpoVS proteins found in sporulating *Firmicutes* species as well as in the non-sporulating *Thermus*-*Deinococcu*s and *Thermotoga* groups, but not in non-sporulating *Firmicutes* species. Phylogenetic reconstruction indicates that *D. turgidum* SpoVS is most closely related to the *D. thermophilum* SpoVS, (Figure 7) followed by the SpoVS of *Thermosediminibacter oceani* DSM 16646. These three SpoVS molecules form a separate clade from the SpoVS of the *Firmicutes* and *Deinococcus/Thermus* species. The SpoVS orthologs show much higher homology than orthologs of other *Dictyoglomus* proteins, suggesting an important conserved function. *Thermotoga* SpoVS orthologs have 53–78% identity, a *Clostridium thermocellum* ortholog has 74% identity, *Caldicellulosiruptor* orthologs have 65–71% identity, and *Thermus* orthologs have 56% identity to Dtur_0800. SpoVS may be an important regulator of cell morphology and differentiation in both sporulating thermophiles where it regulates the transition from vegetative growth to spore formation as well as the non-sporulating thermophiles where it regulates the transition from vegetative growth to formation of multiple morphologies (Brumm P. J. et al., 2015).

## Discussion

We report here the genome sequence, sequence analysis, and cloning of key enzymes of *D. turgidum*, an anaerobic, thermophile reported to degrade a wide range of biomass components including starch, cellulose, pectin and lignin [14]. This hyperthermophile has a small 1.8 M bp genome with a G+C content of the 34%. COGS analysis shows the organism is enriched in genes coding for carbohydrate transport and metabolism. While *Dictyoglomus* make up 25% of the species identified by 16S rRNA sequencing in some environments currently only two species, *D. thermophilum* (Saiki et al., 1985) and *D. turgidus* (Svetlichny and Svetlichnaya, 1988), corrected to *D. turgidum., D. turgidum*, and *D. thermophilum*, have been sequenced and annotated. This first comparison of the two genomes shows that, while the two organisms are unique species, they show extremely high levels of orthologous genes, average nucleotide identity, and synteny. No unique metabolic pathways are present in either organism. Approximately 1/3 of the proteins present in Dtur have orthologs in *D. thermophilum* with over 90% amino acid identity, and less than 10% of the proteins present in either genome have no ortholog in the other genome. The two organisms show extensive short-range and long-range synteny. Genome sequences of additional *Dictyoglomus* species are needed to determine if this is coincidence or a conserved feature of the *Dictyoglomi*. Additional work is also needed to confirm that the differences in synteny observed between the two genomes are real and are not artifacts of the assembly of the genomes.

The genome of *D. turgidum* provides insights into an organism that is strangely foreign and vaguely familiar at the same time. At first glance, the genome is remarkably unremarkable, containing no novel pathways or secondary products. In fact, the organism is lacking many of the pathways normally associated with microbes, including amino acid and fatty acid degradation pathways and energy harvesting via proteins containing hemes, sirohemes, or quinones and appears to be the genome of a strict carbohydrate fermentor. Yet, at the same time, *D. turgidum* cannot be identified as similar to any one organism or Phylum. Results presented here and elsewhere (Nishida et al., 2011; Vesth et al., 2013) show the chameleon-like nature of the organism. Changing the method of comparison radically changes the resulting relationships between *D. turgidum* and other organisms.

The metabolic reconstruction based on the *D. turgidum* genome reveals two unusual features. Most evident is the importance of carbohydrate metabolism for the organism, because *D. turgidum* lacks the ability to metabolize fatty acids and most amino acids. The genome, while lacking in enzymes to degrade crystalline cellulose, possesses genes coding for utilization of most other biomass-derived polymers including xylans, glucans, pectins, arabinans and galactans. Utilization of these polysaccharides appears to involve secretion of enzymes that degrade the polysaccharides to oligosaccharides, transport of the oligosaccharides into the cytoplasm, and degradation of the oligosaccharides to monosaccharides in the cytoplasm. A similar strategy is utilized by thermophilic *Geobacillus* species (Brumm P. et al., 2015). Genes for utilization of carbohydrates are distributed randomly throughout the *D. turgidum* genome, unlike the *Geobacillus* genomes, where genes for individual polysaccharide degradation pathways are organized into distinct operons. The second feature is the obligate fermentative nature of *D. turgidum*. Unlike many other thermophilic anaerobes including *Thermus, Geobacillus, Caldicellulosiruptor*, and *Thermotoga* species, *D. turgidum* possesses no genes for production or utilization of either cytochromes or quinones. Dtur is predicted to utilize the EMP pathway, to produce ATP, reducing equivalents, and fermentation products from monosaccharides. The predicted fermentation products, lactate, acetate, ethanol and hydrogen are in agreement with the published microbiological studies (Svetlichny and Svetlichnaya, 1988) showing the organism produces these four products during fermentation on sugars. The proton gradient needed for ATP generation is produced by NADH oxidoreductase and succinate dehydrogenase, and the ATP is generated by an F0F1-type and a V-type ATP synthases.

Sixteen *D. turdigum* carbohydrases were cloned, expressed and characterized to better understand their function in the metabolism of the organism. The 16 included two each of GH1 and GH3, four GH5, two GH10, and one each of GH36, GH42, GH43, GH53, GH57, and GH67. Based on the proposed mechanism for polysaccharide utilization, *D. turdigum* produces oligosaccharides using secreted enzymes, and degrades the oligosaccharides using intracellular enzymes. The secreted enzymes would be expected to have high substrate specificity to generate oligosaccharides recognized by the transporter systems. The cloned enzymes predicted to be secreted showed activity only on one or two substrates, showing activity on xylan, arabinan, *beta*-glucan, starch, or mannan. Conversely, the intracellular enzymes would be expected to have low specificity, allowing them to degrade multiple substrates and linkages efficiently. The cloned intracellular enzymes typically showed a broader range of activities. GH1 and GH3 enzymes possess *exo*-activity on four or five different carbohydrate substrates. The cloned intracellular xylanase and cellulase both possess both *exo*-activity and *endo*-activity, as well as activity on multiple substrates.

Replication, recombination, and repair enzymes are critical to the genome maintenance and integrity of all cells. Many proteins from this COG functional category are expected to share conserved domains and motifs that could in theory be used to understand the phylogenetic relationship of *D. turdigum* to other organisms. The 16S rRNA genes of *D. turgidum* and *D. thermophilum* share 99% sequence identity. The fraction of replication proteins having 100–90% similarity between the two species is 35%, with 42% sharing 89–80% similarity, 17% sharing 79–70% similarity and 5% with similarity below 69%. The similarity of *D. turgidum* replication proteins to other taxa drops off considerably from that with *D. thermophilum*. The fraction of replication proteins having 100–90% similarity between *D. turgidum* and the next nearest neighbor species is 0%, with 1% sharing 89–80% similarity, 3% sharing 79–70% similarity, 13% sharing 69–60 similarity, 32% sharing 59–50% similarity, 28% sharing 49–40 similarity, and 32% similarity below 40%. This informal comparison again demonstrates how unique *Dictoglomi* are compared to other species. The number and type of replication proteins found in *D. turdigum* is similar to those found in other hyperthermophilic bacteria using IMG tools at the Joint Genome Institute (data not shown). The phylogenetic position of the reverse gyrase does not conform to the 16S rRNA phylogeny suggesting that lateral gene transfer may have taken place.

## Author contributions

FR analyzed data and contributed to manuscript preparation. PB wrote the manuscript, produced and purified Dtur proteins, and performed carbhohydrase analyses. KG produced and purified Dtur proteins. DM managed genome sequencing and analysis, performed DNAP analyses, and contributed to manuscript preparation.

## Funding

This work was completely funded by the DOE Great Lakes Bioenergy Research Center (DOE BER Office of Science DE-FC02-07ER64494 and DOE OBP Office of Energy Efficiency and Renewable Energy DE-AC05-76RL01830). FR acknowledges support from the NASA Exobiology Program.

### Conflict of interest statement

At the time this work was performed, the authors PB and DM were employees and shareholders of C5-6 Technologies Inc. (WI, USA), a company that created bio-based solutions to efficiently convert biomass into five and six carbon sugars. The company ceased operation in December of 2014. PB has since purchased the assets of the company and started C5-6 Technologies LLC (WI, USA), a company focused on supplying reagent enzymes for carbohydrase research. The authors have no other relevant affiliations or financial involvement with any organization or entity with a financial interest in or financial conflict with the subject matter or materials discussed in the manuscript apart from those disclosed. No writing assistance was utilized in the production of this manuscript. The other authors declare that the research was conducted in the absence of any commercial or financial relationships that could be construed as a potential conflict of interest.
